# Small Regulatory RNAs of *Rickettsia conorii*

**DOI:** 10.1038/srep36728

**Published:** 2016-11-11

**Authors:** Hema P. Narra, Casey L. C. Schroeder, Abha Sahni, Mark Rojas, Kamil Khanipov, Yuriy Fofanov, Sanjeev K. Sahni

**Affiliations:** 1 Department of Pathology, The University of Texas Medical Branch, Galveston, TX, 77555, USA; 2 Department of Pharmacology, The University of Texas Medical Branch, Galveston, TX, 77555, USA

## Abstract

Small regulatory RNAs comprise critically important modulators of gene expression in bacteria, yet very little is known about their prevalence and functions in *Rickettsia* species. *R. conorii*, the causative agent of Mediterranean spotted fever, is a tick-borne pathogen that primarily infects microvascular endothelium in humans. We have determined the transcriptional landscape of *R. conorii* during infection of Human Microvascular Endothelial Cells (HMECs) by strand-specific RNA sequencing to identify 4 riboswitches, 13 trans-acting (intergenic), and 22 cis-acting (antisense) small RNAs (termed ‘*Rc*_sR’s). Independent expression of four novel trans-acting sRNAs (*Rc*_sR31, *Rc*_sR33, *Rc*_sR35, and *Rc*_sR42) and known bacterial sRNAs (6S, RNaseP_bact_a, ffs, and α-tmRNA) was next confirmed by Northern hybridization. Comparative analysis during infection of HMECs vis-à-vis tick AAE2 cells revealed significantly higher expression of *Rc*_sR35 and *Rc*_sR42 in HMECs, whereas *Rc*_sR31 and *Rc*_sR33 were expressed at similar levels in both cell types. We further predicted a total of 502 genes involved in all important biological processes as potential targets of *Rc*_sRs and validated the interaction of *Rc*_sR42 with *cydA* (cytochrome d ubiquinol oxidase subunit I). Our findings constitute the first evidence of the existence of post-transcriptional riboregulatory mechanisms in *R. conorii* and interactions between a novel *Rc*_sR and its target mRNA.

Over the past decade, bacterial post-transcriptional regulation is garnering significant interest due to discoveries related to the roles of small RNAs in modulating gene expression during growth and stress conditions *in vitro* and host interactions *in vivo*^1^,^2^. Bacterial small RNAs (sRNAs) are generally 50–500 bp long and fall into three categories, namely riboswitches which are located upstream of mRNAs, *cis-acting* sRNAs synthesized from the complementary strand of an open reading frame (ORF), and *trans-acting* sRNAs transcribed from the intergenic regions with only partial complementarity to their target genes. In contrast to eukaryotic microRNAs which only down-regulate their target mRNAs, bacterial sRNAs can both activate or inhibit translation by either stabilizing the mRNA and opening the ribosome binding site or by degrading the target mRNA, respectively^3^,^4^.

Mediterranean spotted fever (MSF) caused by *Rickettsia conorii* is an acute, febrile illness transmitted to humans through the bite of infected tick(s). *R. conorii* is a Gram-negative obligate intracellular bacterium exhibiting tropism for microvascular endothelium of the blood vessels in mammalian hosts^5^,^6^. Acquisition of a less virulent, dormant phenotype in infected ticks allowing for transovarial transmission to the progeny and transstadial transmission to the next stage during the ticks’ natural developmental lifecycle supports rickettsial persistence, survival, and maintenance in the arthropod vector. In contrast, infection of humans as the dead-end hosts is associated with significant morbidity/mortality attributed to a virulent phenotype^7^. Despite the disease prevalence and significant impact of MSF on public health, little is known about the mechanisms by which *R. conorii* adapts to different host environments and manifests serious disease sequelae such as ‘rickettsial vasculitis’ in the human host while persisting in its arthropod vector.

The seminal publication of the entire genome sequence for *R. prowazekii,* a typhus group *Rickettsia* species, revealed an AT-rich, highly reduced genome, presence of mobile elements and pseudogenes, low coding density compared to the genomes of other intracellular bacteria, and a close evolutionary relationship to eukaryotic mitochondria^8^. A number of other rickettsial genomes belonging to different species have since been sequenced*. R. conorii* genome harbors a single chromosome of 1268755 bp encoding for 1578 protein coding genes, 9 pseudogenes, 33 tRNAs, 2 rRNAs, and contains ~32% intergenic region^9^,^10^. This relatively high percentage of non-coding region in *R. conorii* and other rickettsial genomes has traditionally been considered to be the ‘junk DNA’ or defunct genes resulting from reductive evolution and pseudogenization^7^,^11^,^12^,^13^,^14^. However, recent advances in computational bioinformatics and bacterial molecular genetics have led to the appreciation that the intergenic regions, in addition to harboring transcription factor binding sites and mobile genetic elements, encode for small non-coding RNAs involved in the regulation of target genes. Indeed, the discovery of sRNAs has been a major cornerstone of investigations pertaining to their importance in almost every aspect of bacterial physiology, including pathogenesis, quorum sensing, developmental programming, and regulation of growth and replication. Accordingly, bacterial sRNAs are now well recognized as critically important post-transcriptional regulators in both free-living as well as pathogenic bacteria^3^.

In a recent study, we predicted the existence of ~1700 sRNAs in 13 different species of rickettsiae across all four groups, namely ancestral, spotted fever, transitional, and typhus, and confirmed the expression and biogenesis of six sRNAs in *R. prowazekii*^15^. The specific aims of this follow up study were to identify and catalogue *R. conorii* sRNAs expressed during host cell infection, to understand their conservation profile in different rickettsial species, to compare their expression during infection of human microvascular endothelium vis-à-vis tick vector cells as the host, and to demonstrate the potential riboregulatory roles of trans-acting sRNAs. Using an RNA-Seq based approach, we have identified 39 novel non-coding RNAs across the *R. conorii* chromosome in addition to four well-known bacterial sRNAs (ssrS, ssrA, RNaseP_bact_a, and ffs). Among these, two of the abundantly expressed candidate sRNAs, namely *Rc*_sR35 and *Rc*_sR42, display significant differences in their expression during human *versus* tick cell infection, whereas another two (*Rc*_sR31 and *Rc*_sR33) exhibit similar levels of expression. We have further predicted 502 target genes and 191 gene ontology (GO) functional categories that may be potentially regulated by newly identified trans-acting sRNAs and obtained evidence for *Rc*_sR42 interactions with *cydA* mRNA, implicating regulation of target mRNA transcripts by sRNAs in *R. conorii*. Finally, we have also identified four riboswitches upstream of hypothetical proteins with unknown functions and cis-acting sRNAs corresponding to important, functionally characterized rickettsial genes such as *rickA* and *virB10*, known to be involved in actin-based motility and type IV secretion system, respectively.

## Results

### Identification of novel riboswitches, cis- and trans-acting sRNAs

**Figure 1:**
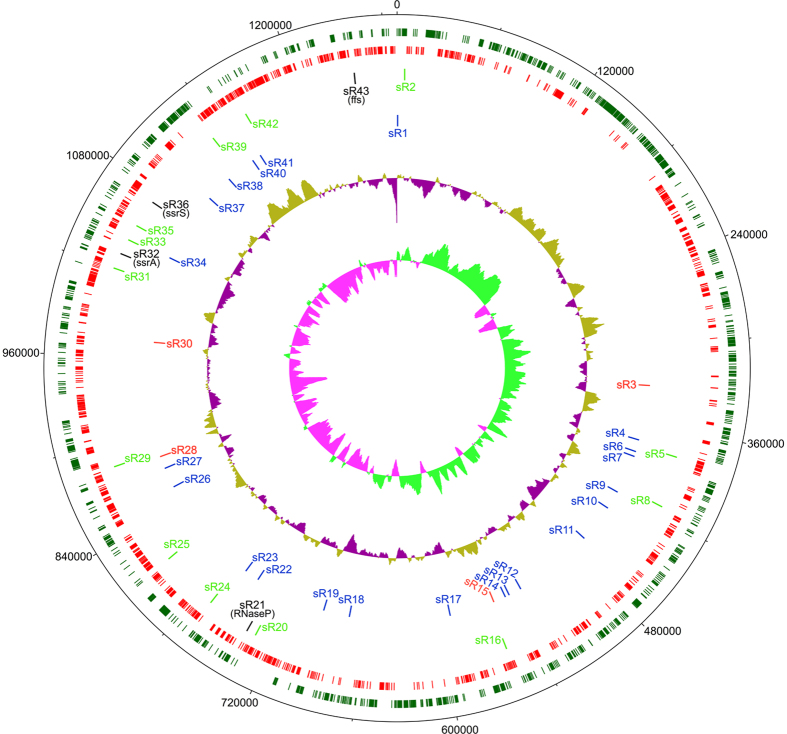
Circular chromosome map of *R. conorii* genome showing the location of four well-known and all sRNAs expressed during the infection of HMECs *in vitro*. The *Rc*_sRs are dispersed throughout the *R. conorii* genome. Complete *R. conorii* genome showing the location of all trans- and cis-acting *Rc*_sRs expressed during the infection of HMECs. The different circles with bars represent: (1) Dark green (outermost): *R. conorii* ORFs annotated on the sense strand, (2) Red: *R. conorii* ORFs annotated on the anti-sense strand, (3) Bright green: Genomic location of novel trans-acting sRNAs, (4) Blue: Genomic location of novel cis-acting sRNAs, (5): GC plot, (6) GC skew (innermost circle). The Black lines in the third circle (outside to inside) represent the 4 well known sRNAs namely, ssrS, ssrA, RNaseP_bact_a, and ffs. The red lines in the fourth circle (outside to inside) represent riboswitches namely, *Rc*_sR3, *Rc*_15, *Rc*_sR28 and *Rc*_sR30. The genome map was generated in dnaplotter using *R. conorii* annotated genome (PATRIC genome ID: 27944.4).

To explore the entire spectrum of *R. conorii* sRNAs expressed during the infection of human microvascular endothelial cells (HMECs), cDNA libraries from total cellular RNA subjected to enrichment for bacterial RNA were processed for Illumina sequencing resulting in an average of 23.76 and 22.20 million paired-end reads at 3 and 24 h post-infection, respectively. To avoid interference due to eukaryotic RNAs, all reads mapping to human genome version 38 were first eliminated and remaining unmapped reads were included in our analysis. To ensure quality control, only the reads with a Phred score of >15 were mapped to complete *R. conorii* genome (PATRIC Genome ID: 272944.4). Thus, approximately 7.26 and 14.29 million reads, accounting for 31% and 64% of the entire read sets at 3 and 24 h post-infection, respectively, mapped to the rickettsial genome (Supplementary Table S1). Among these, there was abundant expression of known bacterial sRNAs, namely, 6S, α-tmRNA, RNaseP_bact_a, and ffs (Supplementary Fig. S1). Importantly, we further identified 22 cis-acting sRNAs on the anti-sense strand of corresponding coding genes, 13 trans-acting sRNAs in the intergenic spacer regions, and four riboswitches within the 5′ leader regions of hypothetical proteins (Fig. 1 and Table 1). The novel cis- and trans-acting sRNAs as well as riboswitches thus identified are hereafter referred to as ‘*Rickettsia conorii*_small RNAs’ (*Rc*_sR).

**Figure 2:**
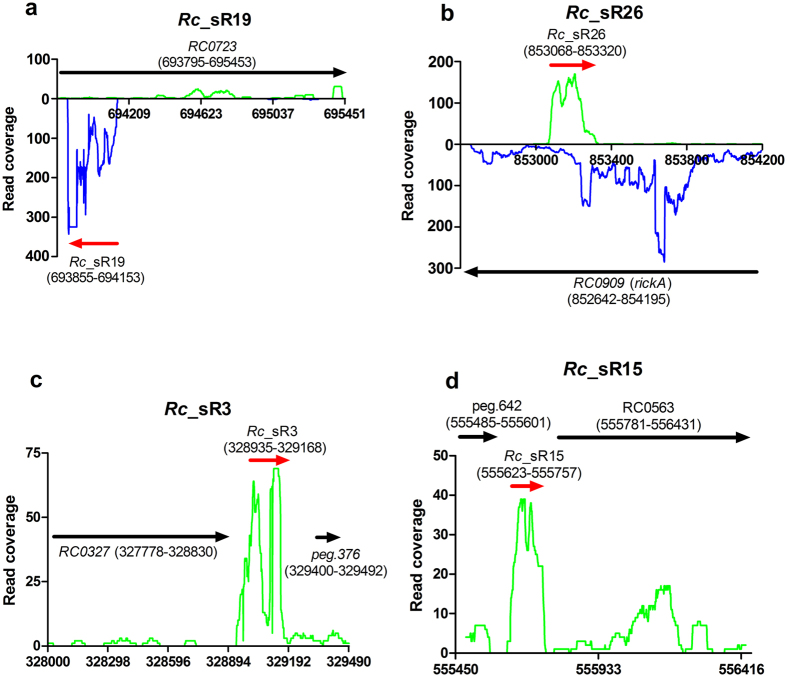
Representative graphs showing the read coverage of novel sRNAs in *R. conorii* during the infection of HMECs. HMECs were infected with *R. conorii* (MOI = 20) and total RNA was harvested at 3 and 24 h post-infection. High throughput RNA sequencing was performed on total RNA enriched for bacterial transcripts (see materials and methods). The strand specific reads were mapped onto *R. conorii* genome (PATRIC genome ID: 27944.4). The read coverage plots of two cis-acting sRNAs (**a**) *Rc*_sR19 and (**b**) *Rc*_sR26, and two riboswitches (**c**) *Rc*_sR3 and (**d**) *Rc*_sR15 are presented. The reads above and below the x-axis represent the forward (green) and reverse strand (blue), respectively. The identified sRNAs are indicated by red arrow. The ORFs up- and downstream, and cis-acting ORF are shown by black arrow. The orientation and genomic location coordinates correspond to the *R. conorii* genome annotation in PATRIC.

All novel sRNAs were found to be expressed as independent transcripts and exhibited an MEV of >2 when compared to 50 nucleotides up- and downstream of the sRNA transcript. As expected, their length ranged from 100 to 400 bp and the average length of cis- and trans-acting sRNAs was 213 bp and 209 bp, respectively (Table 1). No candidate rickettsial sRNA was longer than *Rc*_sR1 (373 bp) and *Rc*_sR38 (120 bp) was the smallest. Also, a majority of both cis-acting (20/22) and trans-acting (12/13) sRNAs in *R. conorii* ranged between 100–300 bp. No significant difference in strand bias was evident since 12 cis-acting and 7 trans-acting sRNAs were located on the anti-sense strand, while the remaining sRNAs were present on the positive strand (Table 1). The genomic context and location of all trans-acting sRNAs was next ascertained by comparing the orientation of up and downstream ORFs with respect to the transcribed sRNA. Seven (*Rc*_sR5, *Rc*_sR8, *Rc*_sR16, *Rc*_sR24, *Rc*_sR25, *Rc*_sR33, and *Rc*_sR42) out of 13 trans-acting sRNAs were transcribed in the opposite orientation with respect to both adjacent genes, and one each was processed in the same direction as their respective upstream (*Rc*_sR2) and downstream (*Rc*_sR35) gene (Table 1). The remaining 4 sRNAs (*Rc*_sR20, *Rc*_sR29, *Rc*_sR31, and *Rc*_sR39) and their respective adjacent up and downstream genes were located on the same strand and transcribed in the same orientation (Table 1). In addition, four riboswitches (*Rc*_sR3, *Rc*_sR15, *Rc*_sR28 and *Rc*_sR30) were positioned upstream of hypothetical proteins. As expected, most of the cis-acting sRNAs originated from the anti-sense strand of an ORF (Table 1), except for *Rc*_sR34 that was found to be overlapping two ORFs (*RC1121* and *RC1122*) as well as the intergenic region between these ORFs (Table 1 and Supplementary Fig. S2). Representative read coverage plots of cis- acting *Rc*_sRs and riboswitches are presented in Fig. 2.

BLAST analysis revealed the presence of sequences homologous (>90% query coverage and identity) to a majority of *R. conorii* trans-acting sRNAs in other spotted fever *Rickettsia* species. Of note, although *Rc*_sR35 was conserved among spotted fever rickettsiae in general, *R. rickettsii* displayed only partially conserved sequence with 47% query coverage. Also, the first 44 bases of *Rc*_sR31 exhibited low level similarity despite excellent overall homology in several SFG rickettsiae. None of the *R. conorii* sRNAs were found to have homologs in typhus group rickettsiae, but the homologs of *Rc*_sR15, *Rc*_sR16, *Rc*_sR29, and *Rc*_sR31 were present with >90% query coverage in at least one of the transitional group species, which include *R. felis, R. akari*, and *R. australis*. Intriguingly, we noticed significant sequence homology (90% identity) between *Rc*_sR3 (328935–329168), a potential riboswitch upstream of a hypothetical protein (peg.376), and *Rc*_sR33 (1041477–1041770), a trans-acting sRNA (Supplementary Fig. S3). In-depth analysis of the genomic context, orientation, and read depth revealed that *Rc*_sR3 was located in the 5′ leader region of peg.376 and transcribed from the forward strand, whereas *Rc*_sR33 was encoded on the reverse strand and located in the intergenic region between RC1120 and RC1121. The average number of *Rc*_sR33 reads were >3-fold higher than those for *Rc*_sR3.

In contrast to trans-acting sRNAs, ORFs coding for all cis-acting sRNAs in *R. conorii* were present in a majority of rickettsial species across different groups and most (17/22) of these ORFs had known function. Notably, genes involved in key functions such as actin based motility (*rickA*), type IV secretion system (*virB10*), bicyclomycin resistance (*bcr1*), cell division (*ftsY*), and LPS biosynthesis (*IpxB*), were identified as possible regulatory targets of cis-acting sRNAs. Only five cis-acting sRNAs were present on the anti-sense strand of hypothetical proteins, some of which may have been independently lost in a few rickettsial species/strains (Supplementary Fig. S4). *Rc*_sR34, spanning across ORFs *RC1121* and *RC1122*, was absent in *R. prowazekii*, but homologous sequences mapped partially to *dnaK* ORF in other *Rickettsia* species, including *R. typhi* (Supplementary Fig. S5). *RC1122*, coding for a 60 amino acid long hypothetical protein in *R. conorii*, exhibited partial sequence homology to full length *dnaK* in other species. Based on genome organization in the *RC1121*-*RC1122* locus, which is unique in *R. conorii* genome with only limited homology to *dnaK* in other rickettsiae, it appears that the genesis of two shorter ORFs (*RC1121-RC1122*) and *Rc*_sR34 may be a consequence of gene degradation in this region (Supplementary Figs 2 and 5).

**Figure 3:**
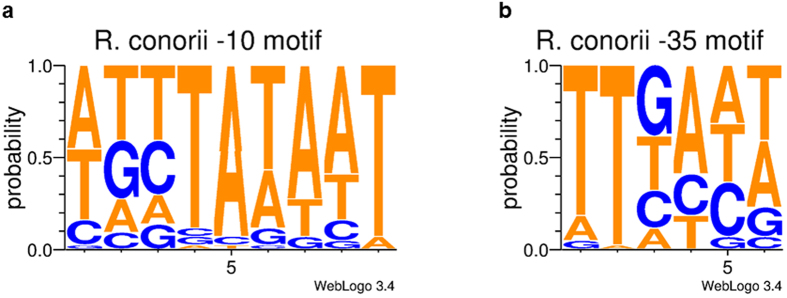
Web logos showing the conservation of −10 and −35 motifs upstream of all novel *R. conorii* sRNAs identified. The 150 bp upstream genomic sequence of all novel *Rc*_sRs identified was subjected to promoter prediction using BPROM^66^. The −10 and −35 motifs were detected upstream of sRNAs and all motif sequences were used to generate consensus sequence based on nucleotide position. The consensus nucleotides for −10 motif (**a**) and −35 motif (**b**) are presented above. A relatively conserved −10 motif was seen upstream of all sRNAs while the −35 motif is less conserved.

The regions upstream of transcriptional start sites (TSSs) of all sRNAs were next analyzed to identify putative promoters and consensus RpoD (σ70) binding sites. All *R. conorii* sRNAs were found to carry a highly consensus −10 box with a typically conserved TATAAT motif that was often preceded by a ‘TT’ dinucleotide in at least 50% of the sRNAs (Fig. 3a). Similar to many other prokaryotes, the −35 motif of sRNAs was relatively less conserved and deviated from the typical *E. coli* housekeeping promoter motif (TTGACA) (Fig. 3b). Interestingly, our attempts to identify *Rc_sRs* with similarity to other known sRNAs in the Rfam non-coding RNA database did not yield any hits, precluding prediction of potential function(s) and/or classification and suggesting that *Rc_sR*s likely represent a unique group of transcriptional regulators in rickettsiae.

### Validation of sRNAs by Northern blot analysis

**Figure 4:**
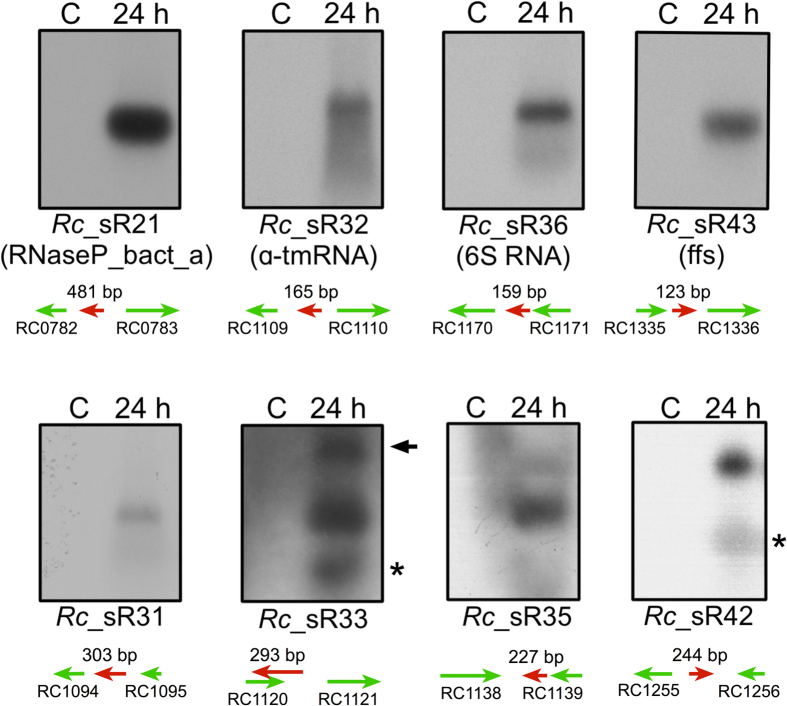
Northern blots showing the expression of selected *R. conorii* novel trans-acting and known bacterial sRNAs during the infection of HMECs. HMECs were infected with *R. conorii* (MOI = 20) and total RNA was extracted at 24 h post-infection. The DNase I treated total RNA was enriched for bacterial transcripts using MICROB*Enrich* and MICROB*Express* kits (Ambion). Enriched RNA was size separated on 1.5% agarose-formaldehyde gel and transferred onto nylon membranes (BioRad). The membranes were probed with [α-^32^P] UTP-labeled strand-specific RNA probes generated by *in vitro* transcription. The membranes were washed following the NorthernMax kit manufacturer’s protocol (Ambion) and developed by autoradiography. All sRNAs were expressed as independent transcripts in *R. conorii* during the infection of HMECs. The scanned images for four novel trans-acting identified in this study (*Rc*_sR31, *Rc*_sR33, *Rc*_sR35 and *Rc*_sR42) and four well-known sRNAs (6S, a_tmRNA, RNaseP_bact_a and ffs) are shown. Two bands of varying transcript sizes were detected in *Rc*_sR33 and *Rc*_sR42 which may represent both primary and processed transcripts. Asterisk indicates a processed transcript of lower size and a non-specific band in *Rc*_sR33 is shown by arrow. The adjacent up and downstream genes of each sRNA are shown in green arrows. The sRNA is indicated by red arrow and the estimated size is shown above the arrow. The orientation of all sRNA and ORFs are based on *R. conorii* genome annotation available in PATRIC. Total RNA from uninfected HMECs was used as a control (C).

To confirm the expression of both novel and known bacterial sRNAs in *R. conorii*, we performed Northern blot analysis. Independent expression of *Rc*_sR31, *Rc*_sR33, *Rc*_sR35 and *Rc*_sR42 was clearly evident during *R. conorii* infection of HMECs (Fig. 4). Interestingly, two transcripts of ~300 and ~250 bp were detected for *Rc*_sR33, of which ~300bp was more prominent, suggesting that smaller transcript of ~250 bp likely represents the processed transcript. In agreement with our RNA-Seq data, abundant and independent expression of *Rc*_sR35 was also seen (Fig. 4). Based on its genomic location and read coverage in RNA-Seq, the transcript size for *Rc_*sR42 was estimated to be ~245 bp. Northern blot analysis demonstrated the presence of two bands of approximately 250 and 200 bp, suggesting that the smaller transcript may be the outcome of either processing or degradation of the primary transcript. For *Rc*_sR31, a single transcript of ~300 bp was also detected in *R. conorii* at 24 h post-infection.

To ascertain the expression of known sRNAs evolutionarily conserved in bacteria, we also determined the expression of 6S, α-tmRNA, RNaseP_bact_a, and ffs in *R. conorii* during infection of HMECs. All sRNAs were expressed as independent transcripts as evidenced by the presence of a single band of the expected size (Fig. 4). Although expression of 6S and RNaseP_bact_a was clearly abundant as shown, relatively lower level of expression of α-tmRNA was observed, which is consistent with the read coverages in our RNA-Seq data (Fig. 4 and Supplementary Fig. S1).

### Expression profile of *R. conorii* sRNAs in human and vector host cells

**Figure 5:**
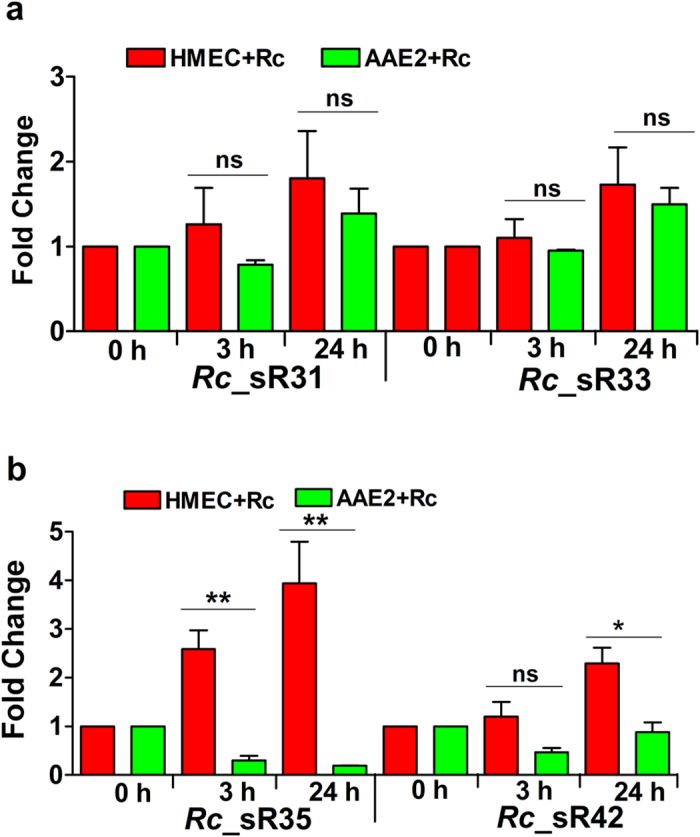
Expression profile of *R. conorii* novel small RNAs, *Rc*_sR31, *Rc*_sR33, *Rc*_sR35 and *Rc*_sR42, during the infection of human endothelium and tick cells *in vitro*. Confluent monolayer of HMECs and AAE2 cells were infected with *R. conorii* (MOI = 20) for 3 and 24 h. Total RNA was extracted by Tri-reagent^®^ method and genomic DNA contamination was eliminated by DNase I treatment. Complementary DNA was generated and the sRNA transcript abundance was assessed by quantitative PCR using sRNA specific primers listed in Supplementary table S2. *R. conorii* 16S rRNA was used as housekeeping control and HMECs or AAE2 cells infected with *R. conorii* for 15 minutes were used as baseline. The data from a minimum of three independent experiments were analyzed by ^∆∆^CT method and presented as mean ± SEM. Significant differences in fold change of *Rc*_sR35 and *Rc*_sR42 were observed depending on the host (bottom panel), while the expression of *Rc*_sR31 and *Rc*_sR33 were similar (top panel) during human and tick cell infection *in vitro*. Legend: Red: Expression of *R. conorii* sRNAs in human cell line (HMECs); Green: Expression of *R. conorii* sRNAs in tick cell line (AAE2). * = p < 0.05, ** = p < 0.005.

To conduct a comparative analysis of *Rc*_sRs in different host niches, we infected HMECs and tick AAE2 cells from *A. americanum* nymphs with *R. conorii* (MOI = 20). Although *Rhipicephalus sanguineus* is the natural vector of *R. conorii, A. americanum* ticks can also acquire and transmit *R. conorii*^16^. All four novel *Rc*_sRs, identified by RNA-Seq and confirmed by Northern blots above, were expressed during the infection of both HMECs and AAE2 cells, indicating their presence and biogenesis in *R. conorii*. In HMECs, the most notable change of ~4 fold up-regulation of *Rc*_sR35 was observed at 24 h post-infection. Expression levels of *Rc*_sR31 and *Rc*_sR33 were apparently higher at 24 h, albeit these differences in transcript levels were not significant (Fig. 5a). Interestingly, *Rc*_sR31 and *Rc*_sR33 showed similar levels of expression in both cell types (Fig. 5a), whereas *Rc*_sR35 and *Rc*_sR42 were expressed at significantly higher levels in HMECs as compared to tick cells (Fig. 5b).

To further ensure the expression of sRNAs as independent transcripts, we next performed qRT-PCR analysis of *Rc*_sR42 and its respective upstream (*RC1255*) and downstream (*RC1256*) ORFs at 3 and 24 h post-infection. Both the flanking genes were highly up-regulated in comparison to *Rc*_sR42 and significant differences in their expression profile were evident clearly indicating that these transcripts are transcribed independently of each other (Supplementary Fig. S6).

### Prediction of *Rc*_sRs’ target genes in *R. conorii*

To determine the functional role(s) of rickettsial trans-acting sRNAs, we employed two independent algorithms, namely IntaRNA and CopraRNA, to identify their target genes^17^ and to further categorize these genes based on their involvement in biological processes such as pathogenesis and virulence using STRING 9.1. Both IntaRNA and CopraRNA, despite identifying a varying number of potential target genes based upon the probability of sRNA-mRNA seed interactions (p < 0.05), predicted several common target mRNAs for each sRNA. The lowest and highest number of targets were predicted to be regulated by *Rc*_sR16 and *Rc*_sR24 (20 *vs.* 53), respectively (Table 2). We further identified a plethora of important biological processes that could be regulated by *Rc*_sR24, including single-organism metabolic and cellular processes, tetrahydrofolate interconversion and metabolic process, response to stress, proteolysis, and other critical cellular and metabolic activities (Supplementary Table S3). In addition, *Rc*_sR31 is predictably involved in the regulation of type IV secretion and protein transport; *Rc*_sR33 is a potential regulator of genes involved in translation and type IV secretion system; the target genes for *Rc*_sR35 are required for porphyrin biosynthesis and heme metabolism; and *Rc*_sR42 may be uniquely involved in RNA and tRNA modification and processing (Supplementary Table S3). Overall, our data reveals that a total of 191 biological processes may be regulated by 13 *R. conorii* sRNAs and regulation of a majority (104) of these functions by only one sRNA. Protein secretion by the type IV secretion system (GO:0030255) is likely regulated by several sRNAs (*Rc*_sR8, *Rc*_sR20, *Rc*_sR31 and *Rc*_sR33). Notably, genes involved in nucleobase-containing compound metabolic process (GO:0006139), protein localization (GO:0008104), protein secretion (GO:0009306), establishment of protein localization (GO:0045184), transport (GO:0006810) and nitrogen compound metabolic process (GO:0006807) are candidates for regulation by 3 different sRNAs indicating their importance in disease pathogenesis and survival mechanisms utilized by *Rickettsia* inside the host cytosol (Supplementary Table S3). Additionally, the predicted secondary fold of 6S sRNA (*Rc*_sR36) exhibits structural similarity to that of *R. prowazekii* and *E. coli*^18^, and the secondary structures of *Rc*_sR31, 33, 35 and 42 display several stems and loops indicating that different regions of the sRNA may be involved in regulating different target genes (Supplementary Fig. S8).

### Validation of sRNA-target mRNA interactions

**Figure 6:**
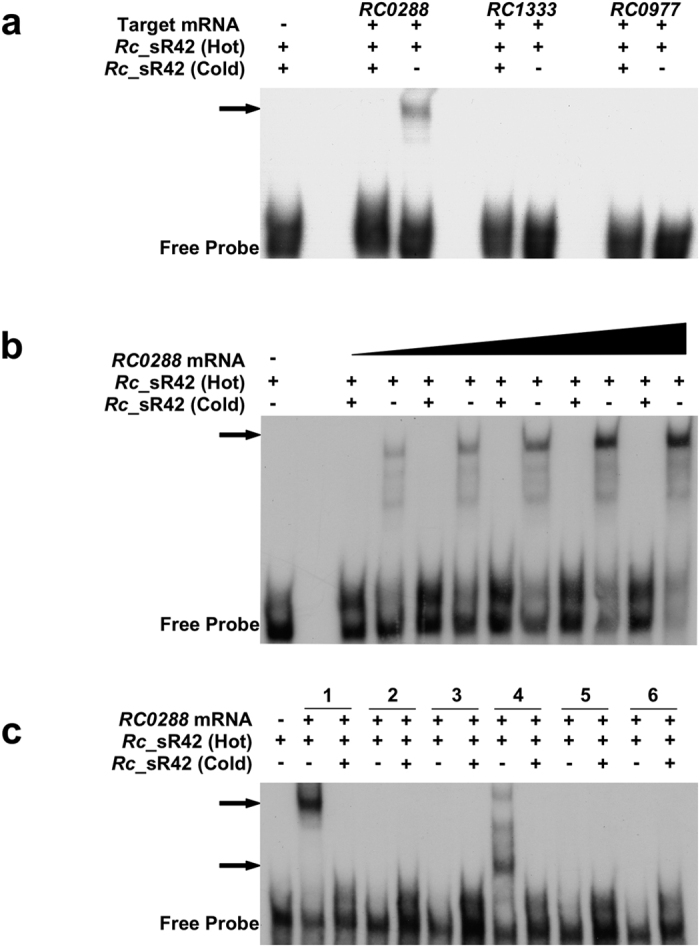
EMSAs showing the interaction of *Rc*_sR42 with *cydA* target mRNA. *In vitro* transcripts of *Rc*_sR42 and target mRNAs were generated from PCR templates amplified from *R. conorii* genomic DNA using primers containing T7 promoter (Supplementary table S2). Only the full length *Rc*_sR42 transcript was labeled with [α-^32^P] UTP. For cold competition, unlabeled full length *Rc*_sR42 was used. The samples were separated on native 4% polyacrylamide gels, vacuum dried, and developed by autoradiography. The photomicrographs shown are a representative gel from three independent experiments. (**a**) EMSA showing the interaction of *R. conorii Rc*_sR42 with *RC0288 (cydA*) target mRNA *in vitro*. The arrow indicates gel-shifted *Rc*_sR42-*cydA* mRNA complex. The other target mRNAs (*RC0977* and *RC1333*) did not show any interaction. (**b**) EMSA showing the interaction of *R. conorii Rc*_sR42 with increasing concentrations of *RC0288 (cydA*) target mRNA *in vitro*. An increase in the complex intensity was observed with increasing concentrations of *RC0288* mRNA. (**c**) EMSA showing the identification of seed region of *Rc*_sR42 and *cydA* interaction. Different regions of *cydA* mRNA fragment (see Supplementary Fig. S9) were used for incubation with full length *Rc*_sR42 to identify the seed region. The genomic locations for *in vitro* transcribed fragments of *cydA* are as follows: 1: 293225–293587 (363 bases); 2: 293284–293587 (304 bases); 3: 293311–293587 (277 bases); 4: 293225–293464 (240 bases); 5: 293284–293464 (181 bases); and 6: 293311–293430 (120 bases). A gel shifted complex (indicated by arrow) was observed only in 1 and 4 indicating that the seed region in *cydA* mRNA is located between bases 293225–293283 corresponding to *R. conorii* genome (PATRIC genome ID: 27944.4).

For experimental validation of target gene predictions, we investigated the interactions between *Rc*_sR42 and four putative target genes, namely, *RC0288 (cydA*), *RC0822 (tlyA*), *RC0977 (grpE*) and *RC1333 (pntAB*). *Rc*_sR42 sRNA was chosen in light of its differential modulation during infection of HMECs versus AAE2 host cells (Fig. 5b) and evidence for its expression as an independent transcript in *R. conorii* (Fig. 4 and Supplementary Fig. S6). Target genes exhibiting significant p value (p < 0.05) for the seed region and encoding conserved proteins such as hemolysin A (*tlyA*), heat shock protein (*grpE*), NAD(P) transhydrogenase α-subunit (*pntAB*), and cytochrome d ubiquinol oxidase subunit I (*cydA*) were chosen to determine riboregulation in *R. conorii*. Gel-shift mobility assays were conducted using *in vitro* generated, isotopically-labeled transcripts of sRNA and target mRNAs in an optimized binding reaction. Interestingly, *Rc*_sR42 formed stable RNA duplex with *RC0288 (cydA*) mRNA (Fig. 6a), which was effectively competed off in the presence of excess unlabeled sRNA, suggesting the specificity of *Rc*_sR42 and *cydA* mRNA interaction. No interactions were observed between *Rc*_sR42 and other chosen target mRNAs (Fig. 6a). To further validate *Rc*_sR42-*cydA* interaction, labeled sRNA was incubated with increasing concentrations of *cydA* mRNA (sRNA to mRNA ratio of 1:1 to 1:20). As shown in Fig. 6b, the intensity of gel shifted complex increased in direct correlation with mRNA concentrations, further ascertaining the specificity and efficacy of target mRNA binding to *Rc*_sR42. Using IntaRNA, we next predicted the seed region (293359–293402) for *Rc*_sR42 binding to be located at the 3′ end of *cydA*. To experimentally authenticate whether the predicted seed region indeed holds true, we systematically performed mobility shift assays using different *cydA* mRNA fragments (schematics presented in Supplementary Fig. S9). Interestingly, the seed region for *Rc*_sR42 interactions with *cydA* mRNA was located slightly upstream of the prediction and between bases 293225 and 293283 of *R. conorii* genome (PATRIC Genome ID: 272944.4) (Fig. 6c).

## Discussion

Reductive evolution owing to progressive gene decay/loss is now widely accepted as a major driving force resulting in smaller genomes in *Rickettsia* species as well as other obligate intracellular bacteria^12^,^19^. Since intracellular pathogens tend to coevolve with their respective hosts, the transcriptional landscape of these organisms varies and several pathways are either independently lost or retained to meet their nutritional and survival requirements depending on the host^19^. For instance, *Rickettsia* lack several important enzymes required for the pathways of amino acid and sugar metabolism, nucleotide synthesis, and lipid biosynthesis, but encode for ATP/ADP translocases to fulfill their energy requirements via exchange of ADP for ATP from the host cytosol^8^,^20^. In contrast, riboflavin synthesis genes in *Buchnera* infer this endosymbiont with the ability to provide riboflavin to its host^21^.

As well-recognized and emerging mediators of gene regulation, bacterial sRNAs are now garnering considerable attention due primarily to their pivotal roles in the transcriptional control of a number of regulatory, enzymatic, and structural mechanisms^1^. Despite the prevalence of sRNAs in many bacteria, there exists only limited evolutionary conservation and both species- and strain-specific sRNA catalogues are now documented^22^. A search of 400 transcriptomic datasets belonging to 40 different strains of bacteria and archaea has revealed that the ‘Goldilocks Zone’ (where species are neither too close nor too distant phylogenetically) for non-coding RNAs is rather narrow, indicating independent evolution of lineage-specific post-transcriptional machinery^23^. As further confirmatory evidence supporting this notion, a search for orthologs of 2208 non-coding RNAs within 1156 bacterial genomes reported in Rfam (a collection of non-coding RNA families)^24^, including members of *Rickettsiales*, also reveals limited taxonomic distribution and suggests a low degree of evolutionary conservation in a majority of these ncRNA families^25^.

To define the non-coding transcriptional landscape of *R. conorii*, we first exploited a global high throughput sequencing approach to identify novel sRNAs expressed during the infection of human endothelial cells *in vitro*. Our underlying rationale here was that to be fully virulent during human infections, vector-borne pathogenic rickettsiae, including *R. conorii,* primarily target microvascular endothelium, rapidly escape from the phagosome, and subvert normal host cell functions to promote their replication and intracellular dissemination. Based on the location of the reads mapping to *R. conorii* genome, four highly conserved (ssrS, ssrA, ffs and RNaseP_bact_a) and 39 (4 riboswitches, 13 trans- and 22 cis-acting) novel sRNAs were identified to be expressed at 3 h (early stage of infection) and 24 h (established infection) post-infection. All sRNAs displayed an MEV of 2-fold or higher in relation to the respective 50 flanking nucleotides, indicating their independent expression, an observation further ascertained by Northern blot analysis and computational identification of σ70 promoters. We have further characterized the expression profile of four trans-acting sRNAs during host-pathogen and vector-pathogen interactions. Importantly, two sRNAs (*Rc*_sR35 and *Rc*_sR42) were differentially expressed in human endothelial cells when compared directly with tick vector cells, suggesting their regulation depending on the host niche. We recently predicted 126 candidate sRNAs to be encoded by *R. conorii* genome using SIPHT/sRNAPredict3, a web-based program based on promoter, transcriptional terminator, and RNA secondary structure prediction tools^15^, but only five of these predicted sRNAs (MEV >2) were identified in this study. It is likely that computational prediction approach reported the presence of potential transcripts based on the conservation of IGRs containing stable RNA secondary structures with defined promoter and terminator sequences, and that several of these predicted sRNAs are either not expressed during infection of host cells or may be expressed under different growth conditions. It is now well appreciated that several bacterial sRNAs are uniquely expressed under conditions of oxygen limitation, iron homeostasis, stress, quorum sensing, and virulence^3^,^26^. For example, sRNA RyhB is highly up-regulated during iron starvation leading to the down-regulation of its target genes *sodB* and *sdhC*/*A* in *E. coli*^27^. More recently, induction and upregulation of PinT, a PhoP activated *Salmonella* sRNA, has been shown to be necessary for transition from invasion to intracellular replication and survival during *in vivo* infection^28^. It is also possible that several sRNA candidates predicted by SIPHT and based on RefSeq annotation, represent unannotated ORFs resulting from differences in genome annotation. A comparison of RAST (Rapid Annotations using Subsystems Technology)^29^ and RefSeq based *R. conorii* genome annotation revealed the presence of 204 ORFs annotated only by RAST, and four SIPHT predicted sRNAs mapped to the genomic location of these uncharacterized ORFs. Thus, in light of prediction *versus* experimental determination, our results validate the use of ‘strand specific deep-sequencing of enriched bacterial transcripts’ approach for identification of novel sRNAs in *R. conorii* and demonstrate its applicability for the evaluation of novel transcripts in other obligate intracellular pathogens.

While some of the *R. conorii* IGRs encoding trans-acting sRNAs share limited homology in other species belonging to the SFG, the ORFs harboring cis-acting sRNAs are conserved in all rickettsial groups including typhus. An intriguing finding in this study is the presence of a riboswitch (*Rc*_sR3) and a novel trans-acting sRNA (*Rc*_sR33) sharing >90% sequence homology (Supplementary Fig. S3). Independent expression of *Rc*_sR33 was confirmed by Northern blot analysis and similar levels of induction were evident during the infection of human endothelial and tick cells *in vitro* (Figs 4 and 5a). Further comparative analysis of *R. conorii* and other rickettsial genomes revealed that only 6 genomes (*R. conorii, R. parkeri, R. slovaca, R. africae, R. peacockii,* and *R. amblyommii*) carry both of these homologous IGRs in their genomes. Interestingly, *RC1120* as an ORF upstream of *Rc*_sR33 with partial overlap with the 3′ end of this sRNA was present only in the genomes harboring both the riboswitch and the trans-acting sRNA. Furthermore, the location of *Rc*_sR3 was upstream of an ORF (peg.376) annotated only by RAST (PATRIC), but not RefSeq (NCBI). The synteny of genomic region adjacent to *Rc*_sR3 was highly conserved, but greater divergence was observed in the genomic location harboring *Rc*_sR33, indicating that *Rc*_sR33 might have originated and coevolved with *RC1120* ORF in few rickettsial genomes (Supplementary Fig. S7). It is rather intriguing that *R. conorii* expresses two unique sRNAs with significant homology, of which one likely functions as a riboswitch upstream of a short ORF (peg.376) and the other is confined to an intergenic region as a trans-acting sRNA. *Pseudomonas aeruginosa* encodes PrrF1 and PrrF2, homologs of a Fur-repressed sRNA RyhB with >95% homology. Both of these sRNAs are expressed during iron starvation and their suppression/deletion is required for the regulation of iron metabolism, indicating similarities in their function as well^30^. On a similar note, multiple copies of Pxr sRNA have been reported in *Cystobacter* and it is hypothesized that Pxr paralogs retain functional similarities resulting in the tight regulation of target genes^31^. In contrary, functional divergence in sRNA paralogs/multiple copies has also been reported. *Legionella pneumophila* and few other bacterial genomes have two copies of 6S RNA, a chelator of σ70 RNA polymerase. Of these, while one copy of 6S regulates the similar set of genes as reported in *E. coli*, another copy of 6S RNA has been shown to regulate distant and restricted set of genes^32^.

The inference of this study that sequences homologous to most *R. conorii* trans-acting sRNAs are confined to only a few other rickettsial genomes excluding those from the typhus group, is expected owing to the genetic diversity resulting from gene degradation, transposon mutagenesis, repetitive and insertion sequences, mobile genetic elements, and lateral gene transfer^13^,^33^. Computational comparison of rickettsial genomes reveals that nearly 50% of the genes encoded by each species/strain are unique to its genome with approximately 700 protein coding genes shared across all genomes^11^,^12^. Even greater genetic divergence is evident for *R. felis*, the etiological agent of a typhus-like flea borne rickettsiosis, whose genome is overrun by mobile genetic elements. Despite coding for ~1600–1800 ORFs, only 300 of these belong to the core set of genes shared by other rickettsial species^14^. It has also been suggested that typhus group genomes have a faster divergence rate in comparison to spotted fever group (SFG) species. Specifically, core proteins in typhus species have 2.43 times higher rate of substitution^11^, indicating that noticeable differences in the rate of evolution and extensive genetic diversity among different species is responsible for an altered and unique transcriptional landscape. Furthermore, single nucleotide polymorphisms (SNPs) in promoter regions can result in the loss of transcription as shown in *E. coli* and *Campylobacter jejuni*^22^. *R. rickettsii* strains Sheila Smith (virulent) and Iowa (avirulent), despite sharing 99% sequence identity, have 492 SNPs and 143 deletions between them^34^. A comparison of sRNAs in 27 *E. coli* and *Shigella* genomes reveals that despite sharing a core set of sRNAs, several sRNAs are highly variable, indicating that the secondary loss of sRNAs, but not horizontal gene transfer, may be the reason for variable distribution even among phylogenetically close organisms^35^. Accordingly, we anticipate that although a few IGRs are shared among the SFG species, some sRNAs may potentially be inactivated or lost leading to differences in sRNA repertoire, thus necessitating the need for identification and characterization of sRNAs in a species- and strain-specific manner to better understand their roles in post-transcriptional regulation.

In general, antisense transcription in bacteria varies between 3–50%^36^. For example, while *E. coli* encodes ~20% of antisense sRNAs, only ~1.5% *Salmonella* sRNAs are cis-acting and a much higher proportion of cis-regulatory elements (27%) are reported in the sRNA repertoire of *Helicobacter*^37^,^38^,^39^. In this study, we have identified 22 cis-acting sRNAs originating from the antisense strand of key regulatory genes such as *rickA, virB10, and ftsY* (Table 1, Fig. 2 and Supplementary Fig. S2). *Rickettsia* species belonging to SFG are known to hijack the host actin assembly for cell-to-cell spread. RickA is critical for the activation of host Arp2/3 complex and required for early stages of motility after invasion. The absence or deletion of *rickA* results in erratic movements or non-motile forms^40^. Bacteria encode for several systems to secrete genetic material, metabolites, and proteins into their extracellular milieu. Type IV secretion system, composed of several Vir proteins, is one of the most thoroughly characterized secretory systems and a majority of these proteins, except for VirB5, are present and conserved in all rickettsial genomes^41^,^42^. Sec-TolC is another secretory system that has been reported in rickettsiae and RARP1, an ankyrin repeat protein, is shown to be secreted in a TolC-dependent manner^43^. Interestingly, apart from a cis-acting sRNA as a potential regulator of *virB10*, we have also identified a sRNA antisense to *ftsY*, encoding a signal recognition particle-like protein. FtsY is an essential component of machinery required for the biogenesis and insertion of proteins into the membrane and a role for this protein has been implicated in Sec translocation system^44^. Additionally, based on the prediction of target genes regulated by trans-acting sRNAs, we project the possibility of regulation of *virD4, virB3* and *virB6* by four different trans-acting sRNAs (*Rc*_sR8, *Rc*_sR20, *Rc*_sR31, *and Rc*_sR33) (Supplementary Table S3). Our findings, thus, present a premise for tight regulation of bacterial motility and secretory systems in *R. conorii*. An important consideration in this context, however, is that approximately 44% of top predictions by CopraRNA turn out to be true and the success rates for IntaRNA and TargetRNA as target prediction tools are 28 and 11%, respectively^45^. Experimental verification and repudiation of *R. conorii* genes predicted as potential targets of *Rc*_sRs by such algorithms would, therefore, be necessary and is currently ongoing.

Several bacterial sRNAs are known to contain tandem repeats and other repeat regions that are critical for their regulation. One classical example is *E. coli* CsrB sRNA, present in several bacterial species. It is known to contain 7 repeats and 18 sites required for binding to CsrA mRNA regulating its translation and stability^3^. Repeat regions have been found in both intergenic spacers and ORFs of coding genes present in *Rickettsia* species^13^,^46^. We have identified two trans-acting sRNAs (*Rc*_sR5 and *Rc*_sR8) and one cis-acting sRNA (*Rc*_sR26, anti-sense to *rickA*) harboring repeat regions in their transcripts. Our strand-specific RNA-Seq showed that both *Rc*_sR26 sRNA and *rickA* (corresponding ORF) are abundantly expressed in *R. conorii* during the infection of HMECs (Fig. 2). It is likely that *Rc*_sR26 expression might be a requisite for stabilizing *rickA* mRNA resulting in its translation. In obligate intracellular bacteria, *Wolbachia* trans-acting sRNA *Wsn*RNA-46 is shown to specifically interact with the palindromic sequences in the *murD* ORF resulting in its down regulation^47^. It is, therefore, possible that trans-acting sRNAs with repeat regions in their transcripts may interact with other complementary repeat regions in coding ORFs resulting in regulation of their expression by direct base pairing. Experimental validation of the interactions between *R. conorii* trans-acting sRNAs and target mRNAs is currently under progress and expected to further illuminate sRNA mediated riboregulatory mechanisms in pathogenic rickettsiae.

Bacterial riboswitches are defined as non-coding RNA elements located within the 5′ UTR of mRNA and exert their regulatory control on the downstream gene in a cis-fashion by directly binding to trans-acting ligand(s)^48^. Our results illustrate the presence of four riboswitches in 5′ non-coding regions upstream of hypothetical proteins with as yet uncharacterized functions. Further, secondary structure analysis of *R. conorii* riboswitches (*Rc*_sR3, sR28, and sR30) reveals terminator/anti-terminator hairpin like structures with a central bulge and *Rc*_sR15 exhibits a single hairpin structure closely resembling that of ThiC riboswitch in *Sinorhizobium meliloti*^49^ (Supplementary Fig. S10). Importantly, the 3′ end of *Rc*_sR3 riboswitch is positioned 233 bp upstream of the 5′end of the downstream coding gene (peg.376). Albeit not a common occurrence, the presence of riboswitches >200 nucleotides upstream of the 5′ region of the downstream gene has been documented for several bacterial species, for example three B_12_ riboswitches in *Listeria monocytogenes.* In addition, there is precedence that such riboswitches can regulate trans-acting sRNAs rather than the downstream ORF^50^,^51^.

Although several conserved bacterial riboswitches and their respective metabolites have been identified by sequence comparison, our attempts to identify potential ligands that may interact with *R. conorii* riboswitches revealed no hits, indicating that riboswitches identified in this study are likely unique and species-specific. Recently, application of ‘Term-seq’ has revealed the presence of 18 new riboswitch candidates as determinants of antibiotic resistance in *Bacillus subtilis*^52^.

Small RNA mediated post-transcriptional regulation can result from different modes of action. Among the well characterized sRNAs, ssrS (6S RNA), ubiquitously present in bacterial genomes, is shown to specifically bind to σ70 holozyme resulting in transcriptional regulation of genes containing σ70 promoters during the stationary phase of growth^53^. The ssrA (α-tmRNA) acts by releasing the stalled ribosomes during translation, while ffs (4.5S RNA) is involved in the targeting of proteins to the membranes immediately after translation^54^,^55^. Cis-acting sRNAs, originating from the anti-sense strand of a coding gene potentially bind to their counterparts by base pairing of complementary sequences resulting in transcriptional regulation of the ORF. Complexities of regulation by trans-acting sRNAs have also been reported. In several bacteria, the interactions of trans-acting sRNAs with their target mRNAs are facilitated by RNA chaperones and the location of seed region in the target mRNA determines the fate of the mRNA. In some instances, trans-acting sRNAs may stabilize the transcript by initiation of translation, while in other cases, the target mRNA may be degraded by ribonucleases. Using two independent algorithms, we predict the possibility of regulation of a total of 502 target genes by *R. conorii* trans-acting sRNAs. Key regulatory pathways involved in LPS biosynthesis, nucleotide metabolism, secretion, protein biosynthesis and metabolism, and carbohydrate biosynthesis are predicted to be regulated by these intergenic sRNAs (Supplementary Table S3). Furthermore, we have experimentally validated the interaction of *Rc*_sR42 with *cydA* mRNA (Fig. 6). In *Rickettsia, cydA* encodes for cytochrome d ubiquinol oxidase subunit I, a terminal oxidase required for aerobic respiration. The cytochrome bd oxidase encoded by *cydAB* operon in *Coxiella* is shown to exhibit high affinity to oxygen and is required for ATP synthesis during microaerophilic intracellular growth^56^. The *cydA* and *cydB* are synthesized as polycistronic mRNAs in *R. conorii* and the sRNA-mRNA seed region lies at the 3′ end of the *cydA* transcript at genomic position 293225–293283, slightly upstream of the location predicted by IntaRNA (293359–293402) (Fig. 6). We reason that this may be because predictive algorithms often ignore the sRNA secondary structure complexity, pseudoknots, and double-kissing hairpin complexes^45^. Since sRNA-mRNA interactions tend to occur over a short and imperfect complementarity, further investigations to decipher critical base(s) within the validated 60 bp seed region (293225–293283) are now in progress. The regulatory outcomes of such an interaction may likely be that *Rc*_sR42 is either involved in stabilizing the *cydA* transcript as a result of the cleavage of polycistronic *cydAB* transcript or in the degradation of *cydAB* transcript by forming a double stranded RNA. Rickettsial genomes encode ATP/ADP translocases and can also synthesize ATP during later stages of infection when ATP supply in the host cytosol is exhausted. Intriguingly, *Rc*_sR42 is highly expressed at 24 h post-infection (Fig. 5b), indicating that regulation of *cydA* mRNA at later stages of infection may facilitate *R. conorii* survival in the intracellular niche. Furthermore, *cydA* mutants in several bacteria are unable to survive indicating the key functional role of this protein in survival. Comprehensive molecular studies employing appropriate heterologous model systems and aimed at generating knock-out mutants should reveal the functional implications of such regulatory sRNAs.

Although a role for hfq, an RNA chaperone, in facilitating mRNA interactions with trans-acting sRNAs is well established, the homologs of hfq are absent in rickettsial genomes. As of now, it is not clear if trans-acting sRNAs identified in this study would function in a chaperone-dependent or –independent manner. Hypothetical proteins such as HP1334 in *H. pylori* and several other RNA binding proteins such as YbeY in *Sinorhizobium meliloti* have been implicated with chaperone activity^57^,^58^. More recently, a ProQ/FinO domain containing protein Lpp0148 has been reported to function as a RNA chaperone in *Legionella pneumophila*^59^. It is, therefore, possible that *R. conorii* proteins with unknown function may play a role in sRNA-mediated regulation. At this stage, our preliminary investigations of this aspect reveal that total protein extracts from *R. conorii* enhance *Rc*_sR42 interaction with *cydA,* indicating chaperone-like activity of as yet unidentified rickettsial protein(s) (Narra *et al*., unpublished data). Alternatively, the trans-acting sRNAs may bind to their target mRNA by direct base pairing at the seed regions in a chaperone-independent manner.

In conclusion, our high resolution transcriptomic profiling revealed the presence of novel non-coding RNAs in *R. conorii* during host-pathogen interactions. We have further shown that two trans-acting sRNAs (*Rc*_sR35 and *Rc*_sR42) are differentially expressed during host-pathogen and vector-pathogen interactions, indicating their role in survival and transovarial/transstadial transmission in arthropod vectors and regulation of virulence in the human host. Most notably, we also provide the first experimental evidence for riboregulation in rickettsiae. Investigations are underway to further validate the importance of post-transcriptional regulatory network in the mechanisms of rickettsial survival, adaptation, and pathogenesis.

## Material And Methods

### Preparation of *R. conorii* stocks

*R. conorii* (Malish 7 strain) was grown in cultured Vero cells, purified by differential centrifugation as described previously^60^, and stored frozen at −80 °C as aliquots to avoid repeated freeze-thaw cycles. The infectivity titers of purified stocks were estimated by citrate synthase (gltA)-based quantitative PCR following a published protocol^61^.

### Cell culture and infection

Human microvascular endothelial cells (HMECs) were cultured at 37 °C in MCDB131 medium supplemented with 10% fetal bovine serum, 10 mM L-glutamine, 1 μg/ml hydrocortisone, and 10 ng/ml epidermal growth factor in an atmosphere of 95%O_2_:5%CO_2_. Cells were infected with *R. conorii* at an MOI of 20 following our established protocol^62^. Briefly, cell monolayers were incubated with *R. conorii* in a minimum volume of culture medium to ensure efficient adhesion and internalization. After 15 minutes, cells were placed in fresh medium and incubated at 37 °C for 3 and 24 h. For comparative analysis, the condition in which *R. conorii* was incubated with the host cells for 15 minutes only was designated as the ‘baseline’.

*Amblyomma americanum* tick cells (AAE2) were grown in L-15B complete medium (pH 7.5) at 34 °C as described^63^. The AAE2 cells were infected with *R. conorii* (MOI = 20) at 34 °C for 15 minutes. At this point, the medium was gently aspirated off and centrifuged to collect any viable semi-adherent cells in culture. The pellet was resuspended in fresh L-15B infection medium and added back to the culture flasks for further incubation for 3 and 24 h. Similar to the HMECs above, AAE2 cells infected with *R. conorii* for the first 15 minutes to simply allow sufficient time for adhesion and invasion were employed as the baseline control.

### RNA extraction and library preparation

Total RNA from *R. conorii-*infected HMECs was extracted by Tri-reagent^®^ method^64^. The RNA thus obtained was treated with DNase I (0.5 units/μg RNA) at 37 °C for 1 h to remove any genomic DNA. The RNA quality was then verified for its integrity on an Agilent 2100 BioAnalyzer (Agilent Technologies) and samples with a RIN score of ≥9.0 were used in our experiments. Enrichment for bacterial transcripts was next performed using Dynabeads^®^ Oligo (dT)_25_ (ThermoFisher Scientific) to capture eukaryotic polyadenylated mRNAs and a Ribo-Zero™ kit (Illumina) to remove eukaryotic (18S/28S) rRNAs and bacterial (16S/23S) rRNAs. The enriched RNA was reverse transcribed and subjected to the preparation of strand-specific cDNA libraries using TruSeq RNA Sample Prep Kit (Illumina). A minimum of two independent samples meeting our quality-control criteria were processed for the preparation of cDNA libraries belonging to complementary strands and deep sequencing as outlined below.

### RNA Sequencing and identification of small RNAs

Strand-specific cDNA libraries were sequenced as 50 base long, paired-end reads on Illumina Hi-Seq 1000 at the Next Generation Sequencing core facility, UTMB. The sequencing read statistics for each library are presented in Supplementary Table S1. The reads from each library were analyzed for their base quality and any base with a PHRED score of ≤15 was excluded from the analysis. The reads were then mapped to the complete, annotated genome of *R. conorii* (Malish 7) available in Pathosystems Resource Intergration Center (PATRIC Genome ID: 272944.4) allowing up to two mismatches per read using Bowtie 2^65^. The coverage for each nucleotide was visualized in Integrated Genome Viewer (IGV: Broad Institute) and bacterial sRNAs were identified depending on the origin of reads, i.e. the reads mapping either to the intergenic region (trans-acting) or to the complementary strand of a coding open reading frame (cis-acting), or to the 5′ regions upstream of mRNA (riboswitches). Expression levels were determined by normalizing the number of reads mapping to the genomic region corresponding to the sRNA against total number of reads mapping to *R. conorii* genome (excluding those mapping to rRNAs and known tRNAs). The average expression values from independent libraries were then calculated and designated as the Mean Expression Value (MEV). A small RNA was considered to be bona fide, if its MEV was ≥1.5 fold in direct comparison to the same for 50 flanking nucleotides and it did not correspond to or contain an ORF within the sequence based on *R. conorii* genome in PATRIC. Coverage plots for select sRNAs and their respective up and downstream genes were generated using GraphPad Prism. The presence of consensus sigma-70 (σ^70^) promoters upstream of all small RNAs was identified using BPROM^66^. The minimum free energy based secondary structures of *Rc*_sR31, 33, 35, 36 (6S), and 42 were predicted by RNAfold webserver (http://rna.tbi.univie.ac.at/cgi-bin/RNAfold.cgi) using default parameter settings.

### Quantitative real-time PCR

The primers for real-time PCR (Supplementary Table S2) were designed using Primer Express 3.0.1 (Applied Biosystems). To determine the expression of *R. conorii* sRNAs and their adjacent upstream and downstream genes, total RNA from HMECs and AAE2 cells infected with *R. conorii* was reverse transcribed (1 μg RNA as the input) using high capacity cDNA reverse transcription kit (Life Technologies). Quantitative PCR (qRT-PCR) was performed using SYBR Green-based assay with rickettsial 16S rRNA as the housekeeping control. The Ct values for 3 and 24 h post-infection were normalized to the baseline control, which was assigned a value of 1, and analyzed by ^∆∆^Ct method^64^. The data sets were calculated as the mean ± SEM from a minimum of three independent experiments and statistical analysis was performed using GraphPad Prism with statistical significance set to a threshold *P*-value of ≤ 0.05.

### Northern blot analysis

To confirm the expression of *R. conorii* sRNAs identified in the RNA-Seq analysis, Northern blot analysis was performed using enriched RNA preparations and NorthernMax^®^ kit reagents (Ambion). Briefly, total RNA was enriched for bacterial transcripts using MICROB*Enrich* and MICROB*Express* kits (Ambion) and resultant enriched RNA was size separated on 1.5% formaldehyde-agarose gels and transferred onto positively-charged nylon membranes (Bio-Rad). For hybridization, [α-^32^P] UTP-labeled strand-specific RNA probes were generated by *in vitro* transcription using sRNA-specific primers (Supplementary Table S2) and MAXIscript^®^ kit (Ambion). The radioactively labeled probes were purified using Sephadex microspin columns (GE Healthcare) and used for overnight hybridization according to the NorthernMax^®^ Kit (Ambion). Finally, the blots were exposed to autoradiography films (BioExpress) and images were scanned and saved as TIFF files.

### Prediction and functional enrichment of target genes regulated by trans-acting sRNAs

Two independent programs, IntaRNA and CopraRNA^17^, were used to identify target genes for *R. conorii* sRNAs. The predictions by IntaRNA are based on minimum hybridization energy between two RNA molecules taking the accessibility and length of seed region into consideration, while CopraRNA integrates phylogenetic information to predict sRNA targets at the genomic scale and reconstructs regulatory networks employing functional enrichment and network analysis, allowing for high confidence target prediction and efficient classification of sRNAs. For both programs, default settings were employed with the only exception that the region under interrogation was adjusted to include −150 to +100 base region with respect to the transcription start site of the target gene. All target genes exhibiting significant (p < 0.05) interaction in the sRNA-mRNA seed regions were considered for further analysis. To gain insight into their functional roles, target genes common to both programs and displaying a seed region p-value of <0.05 were used for functional categorization using STRING 9.1^67^ and only the gene ontology (GO) functional categories showing a significant p-value of <0.05 were included in the analysis.

### Electrophoretic mobility shift assay (EMSA)

To validate the binding interactions between sRNA and mRNA, EMSA were performed following a standard protocol^68^. All primers are listed in Supplementary Table S2. Specifically, [α-^32^P]UTP-labeled full length *Rc*_sR42 (1160321-1160565: 245 bp) and target mRNA transcripts were generated by *in vitro* transcription using T7 polymerase and MAXIscript^®^ kit following the manufacturer’s instructions (Ambion). The mRNA fragments used in the study, based on the PATRIC annotation of *R. conorii* genome, were as follows: *RC0288 (cydA*): 293225–293587 (363 bp), *RC0822 (tlyA*): 777540–777819 (280 bp), *RC0977 (grpE*): 915855–916054 (200 bp), and *RC1333 (pntAB*): 1237558–1237877 (320 bp). To identify seed region in the *cydA* fragment used above, fragments of varying legths were generated by *in vitro* transcription. The genomic locations for *in vitro* transcribed fragments of *cydA* were: 293225–293587 (363 bases); 293284–293587 (304 bases); 293311–293587 (277 bases); 293225–293464 (240 bases); 293284–293464 (181 bases); and 293311–293430 (120 bases). The schematic of different *cydA* mRNA fragments used in this study is presented in the Supplementary Fig. S9. The *in vitro* transcribed sRNA and mRNA were mixed in a binding buffer (Promega) and incubated at 70 °C for 5 minutes followed by 30 °C for 15 minutes. Since RNAs are known to exhibit complex structures, incubation at 70–90 °C for 1–5 minutes was found to be optimal to achieve RNA population with homogeneous fold and has been extensively followed while performing EMSA studies to identify the sRNA-mRNA interactions^69^,^70^,^71^. Further incubation at 30 °C for 15 minutes was chosen for this study as *Rickettsia* species while present in tick vectors are exposed to temperatures ranging from 28–30 °C. A five-fold excess of unlabeled sRNA was used to ensure specificity of interactions. The samples were separated by electrophoresis on a native 4% polyacrylamide gel, which was vacuum-dried on a Whatman filter and subjected to autoradiographic exposures. The images were scanned and saved in TIFF format.

## Additional Information

**How to cite this article**: Narra, H. P. *et al*. Small Regulatory RNAs of *Rickettsia conorii. Sci. Rep.* **6**, 36728; doi: 10.1038/srep36728 (2016).

**Publisher’s note:** Springer Nature remains neutral with regard to jurisdictional claims in published maps and institutional affiliations.

## Supplementary Material

Supplementary Information

Supplementary Table S1

Supplementary Table S2

Supplementary Table S3

## Figures and Tables

**Table 1:** List of *R. conorii* sRNAs expressed during the infection of human microvascular endothelial cells *in vitro.*

sRNA^a^	Start^b^	Stop^b^	Size (bp)	Riboswitch/Cis/Transacting^c^	Upstream ORF^d^	Downstream ORF^d^	Cis-ORF^d^	Orientation^e^
*Rc*_sR1	466	838	373	cis	—	—	peg.1 (*RC0001*)	</>
*Rc*_sR2	6026	6239	214	trans	peg.5 (*RC0005*)	peg.6 (*RC0007*)	—	>/>/<
*Rc*_sR3	328935	329168	234	riboswitch	—	peg.376	—	>/>
*Rc*_sR4	351165	351368	204	cis	—	—	peg.395 (*RC0347)*	</>
*Rc*_sR5	379393	379571	179	trans	peg.432 (*RC0377*)	peg.433 (*RC0378*)	—	</>/<
*Rc*_sR6	384964	385196	233	cis	—	—	peg.438 (*RC0383*)	>/<
*Rc*_sR7	389015	389183	169	cis	—	—	peg.444 (*RC0389*)	</>
*Rc*_sR8	414438	414565	128	trans	peg.467 (*RC0412*)	peg.468 (*RC0413*)	—	>/</>
*Rc*_sR9	420477	420741	265	cis	—	—	peg.480 (*TolA*)	</>
*Rc*_sR10	435394	435697	304	cis	—	—	peg.498 (*RC0440*)	</>
*Rc*_sR11	466022	466209	188	cis	—	—	peg.537 (*RC0469*)	</>
*Rc*_sR12	531156	531375	220	cis	—	—	peg.619 (*RC0540*)	>/<
*Rc*_sR13	541604	541785	182	cis	—	—	peg.629 (*RC0550*)	>/<
*Rc*_sR14	545029	545234	206	cis	—	—	peg.632 (*RC0553*)	>/<
*Rc*_sR15	555623	555757	135	riboswitch	—	peg.643 (*RC0563*)	—	>/>
*Rc*_sR16	559197	559344	148	trans	peg.648 (*RC0567*)	peg.649 (*RC0568*)	—	</>/<
*Rc*_sR17	591563	591758	196	cis	—	—	peg.691 (*RC0605*)	>/<
*Rc*_sR18	672763	672934	172	cis	—	—	peg.793 (*RC0698*)	</>
*Rc*_sR19	693856	694153	298	cis	—	—	peg.823 (*RC0723*)	</>
*Rc*_sR20	732978	733122	145	trans	peg.876 (*RC0773*)	peg.877 (*RC0774*)	—	</</<
*Rc*_sR21 (RNaseP_bact_a)	739375	739855	481	—	peg.887 (*RC0782*)	peg.888 (*RC0783*)	—	</</>
*Rc*_sR22	752032	752195	164	cis	—	—	peg.907 (RC0798)	>/<
*Rc*_sR23	763895	764018	124	cis	—	—	peg.918 (*RC0808*)	</>
*Rc*_sR24	770130	770278	149	trans	peg.925 (*RC0815*)	peg.926 (*RC0816*)	—	</>/<
*Rc*_sR25	811219	811387	169	trans	peg.978 (*RC0860*)	peg.979 (*RC0862*)	—	>/</>
*Rc*_sR26	853068	853320	253	cis	—	—	peg.1033 (*RC0909*)	>/<
*Rc*_sR27	869414	869639	226	cis	—	—	peg.1051 (*RC0924*)	>/<
*Rc*_sR28	880969	881142	174	riboswitch	—	peg.1074 (*RC0942*)	—	</<
*Rc*_sR29	883911	884189	279	trans	peg.1077 (*RC0945*)	RCRNA26	—	</</<
*Rc*_sR30	969095	969353	259	riboswitch	—	peg.1196 (*RC1045*)	—	</<
*Rc*_sR31	1020148	1020450	303	trans	peg.1256 (*RC1094*)	peg.1257 (*RC1095*)	—	</</<
*Rc*_sR32 (α_tmRNA, ssrA)	1030837	1031001	165	—	peg.1278 (*RC1109*)	peg.1279 (*RC1110*)	—	</</>
*Rc*_sR33	1041477	1041770	294	trans	peg.1290 (*RC1120*)	peg.1291 (*RC1121*)	—	>/</>
*Rc*_sR34^f^	1042717	1042899	183	cis	peg.1291 (*RC1121*)	peg.1292 (*RC1122*)	—	>/</>
*Rc*_sR35	1052516	1052743	228	trans	peg.1310 (*RC1138*)	peg.1311 (*RC1139*)	—	>/</<
*Rc*_sR36 (6S, ssrS)	1071875	1072033	159	—	peg.1346 (*RC1170*)	peg.1347 (*RC1171*)	—	</</<
*Rc*_sR37	1100103	1100327	225	cis	—	—	peg.1380 (*RC1205*)	</>
*Rc*_sR38	1121923	1122042	120	cis	—	—	peg.1397 (*RC1218*)	</>
*Rc*_sR39	1132389	1132619	231	trans	peg.1406 (*RC1227*)	peg.1407 (*RC1228*)	—	>/>/>
*Rc*_sR40	1146031	1146254	224	cis	—	——	peg.1419 (*RC1238*)	>/<
*Rc*_sR41	1153327	1153508	182	cis	—	—	peg.1428 (*RC1246*)	>/<
*Rc*_sR42	1160321	1160565	245	trans	peg.1439 (*RC1255*)	peg.1440 (*RC1256*)	—	</>/<
*Rc*_sR43 (ffs)	1239529	1239651	123	—	peg.1530 (*RC1335*)	peg.1531 (*RC1336*)	—	>/>/>

^a^*R. conorii* sRNAs are numbered depending on their genomic location in the genome annotation available in PATRIC and starting from the 5′ of the genome. The names in parentheses are the other names reported in the databases for the respective sRNA.

^b^The start and stop co-ordinates for the sRNAs corresponding the *R. conorii* genome annotation available in PATRIC.

^c^Based on the genomic location, the sRNAs are defined as riboswitches or as ‘cis-‘ (present on the anti-sense strand of an ORF) or ‘trans-acting’ (intergenic region).

^d^The upstream, downstream and cis-ORFs are identified based on the *R. conorii* genome annotation available in PATRIC. The number in parentheses indicate known alternative name of the ORF.

^e^The arrows ‘>’ and ‘<’ indicate ‘sense’ and ‘anti-sense’ orientation respectively. For all riboswitches, the orientation of the riboswitch and the downstream ORF, respectively, are shown. For trans-acting sRNAs, the orientation of upstream ORF, sRNA and downstream ORF are shown in order. The orientation of cis-acting sRNAs is shown as sRNA and cis-ORF, respectively.

^f^*Rc*_sR35 is a cis-acting sRNA present on the positive strand and spanning across both up and downstream ORFs (*RC1121* and *RC1122*) coding from the negative strand. So the orientation of upstream ORF, sRNA and downstream ORF are shown.

**Table 2:** List of targets gene regulated by *R. conorii* trans-acting sRNAs.

sRNA	Number of predicted target genes^a^		Number of common target genes predicted by both programs^b^
	CopraRNA	IntaRNA	
*Rc*_sR2	60	56	39
*Rc*_sR5	36	33	21
*Rc*_sR8	61	49	44
*Rc*_sR16	46	30	20
*Rc*_sR20	77	69	41
*Rc*_sR24	71	57	53
*Rc*_sR25	59	45	31
*Rc*_sR29	62	55	32
*Rc*_sR31	65	67	42
*Rc*_sR33	60	66	49
*Rc*_sR35	62	62	48
*Rc*_sR39	74	73	49
*Rc*_sR42	72	53	33

^a^Only target genes having a significant p-value (p < 0.05) for the predicted seed region are included in the analysis.

^b^Targets predicted by both CopraRNA and IntraRNA and having significant p-value (p < 0.05) for the predicted seed region.
