# Supplementary material for: Small Regulatory RNAs of Rickettsia conorii
Source: Sci Rep. 2016 Nov 11;6:36728. doi: 10.1038/srep36728 (PMC5105140; doi:10.1038/srep36728)

Small Regulatory RNAs of *Rickettsia conorii*

Hema P. Narra[1], Casey L. C. Schroeder[1], Abha Sahni[1], Mark Rojas[2], Kamil Khanipov[2], Yuriy Fofanov[2], and Sanjeev K. Sahni[1*]

[1]Department of Pathology, The University of Texas Medical Branch, Galveston, TX, USA 77555.

[2]Department of Pathology, The University of Texas Medical Branch, Galveston, TX, USA 77555.

Supplementary Figures

Supplementary Figure S1: Read coverage plots of four well-known sRNAs expressed in *R. conorii* during the infection of HMECs *in vitro*.
The coverage plots of 6S (ssrS), a_tmRNA (ssrA), RNaseP_bact_a and ffs are shown.

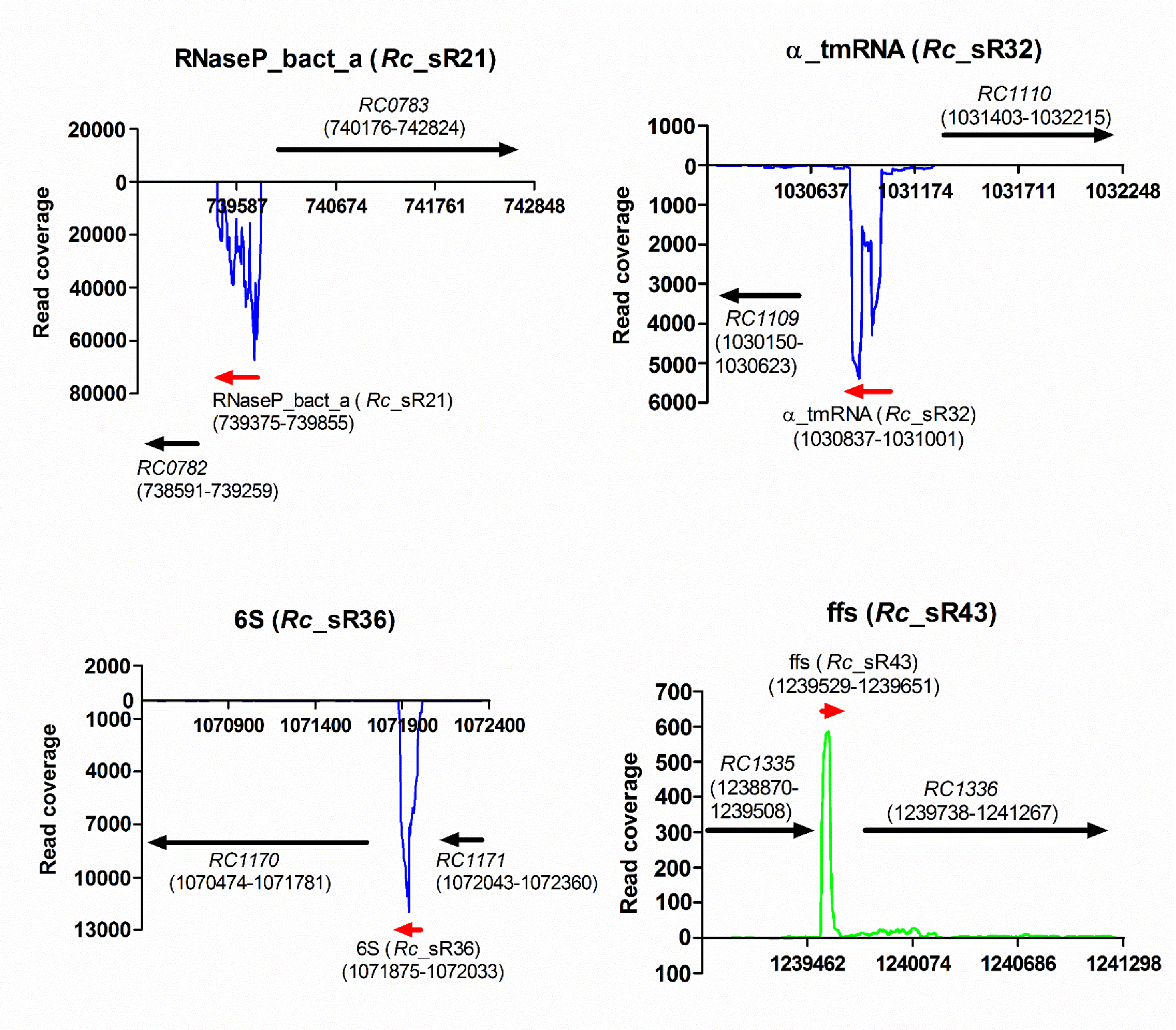

Supplementary Figure S2: Read coverage plots showing the expression of two novel cis-acting sRNAs namely *Rc*_sR7 and *Rc*_sR34 in *R. conorii* during the infection of HMECs.

A: coverage plot of cis-acting sRNA *Rc*_sR7. The cis-ORF (*virB10*) is shown by black arrow and the sRNA is depicted by red arrow. B: coverage plot of cis-acting sRNA *Rc*_sR34. The cis-ORFs (*RC1121* and *RC1122*) are shown by black arrows and the sRNA is depicted by red arrow.

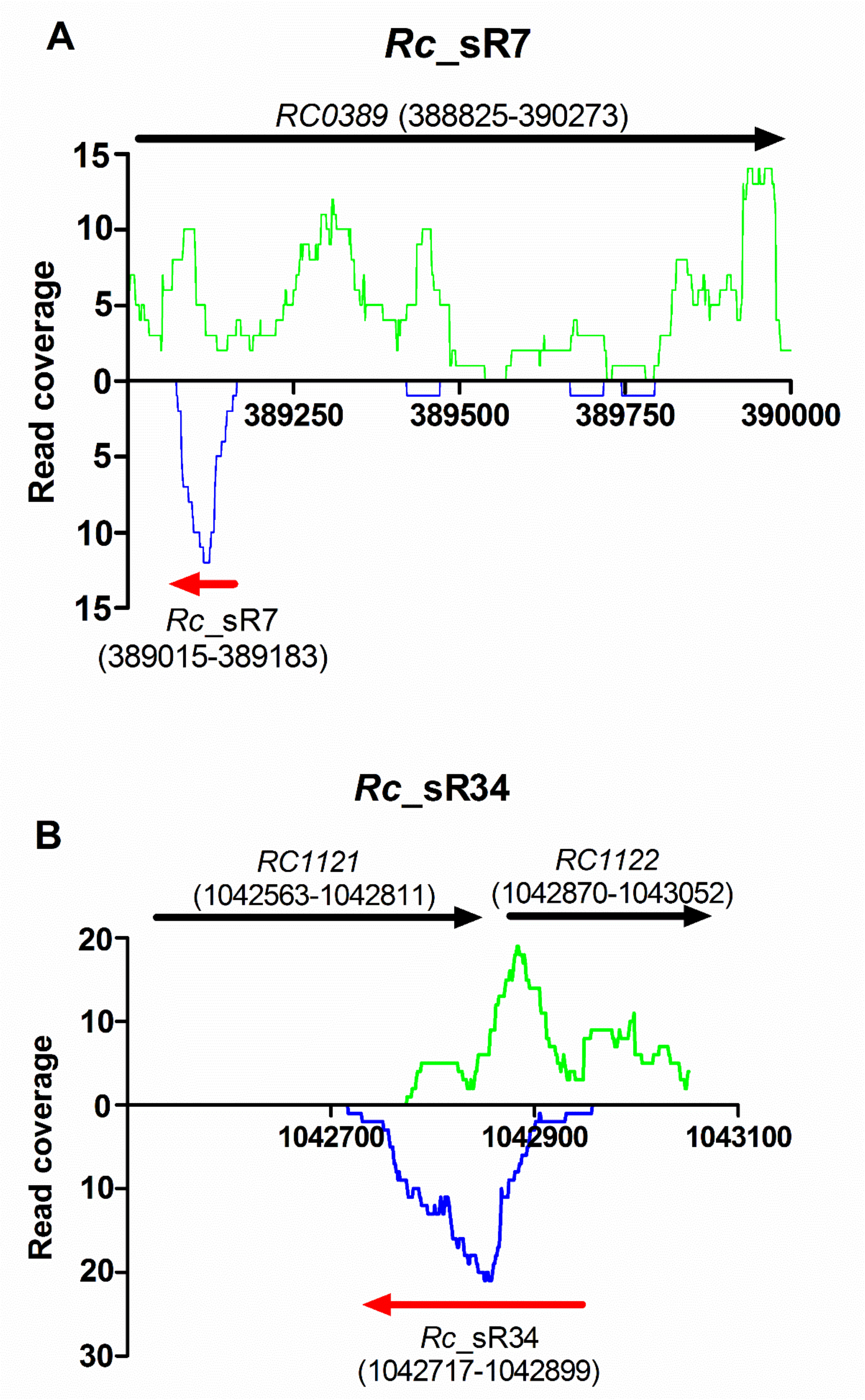

Supplementary Figure S3: Alignment of *Rc*_sR3 and *Rc*_sR33 expressed in *R. conorii* during the infection of HMECs.

The genomic sequences of *Rc*_sR3 and *Rc*_sR33 were downloaded from PATRIC and aligned in CLUSTAL Omega. The *Rc*_sR3 and *Rc*_sR33 are expressed in two different genomic locations in the *R. conorii* and share 90% sequence homology.

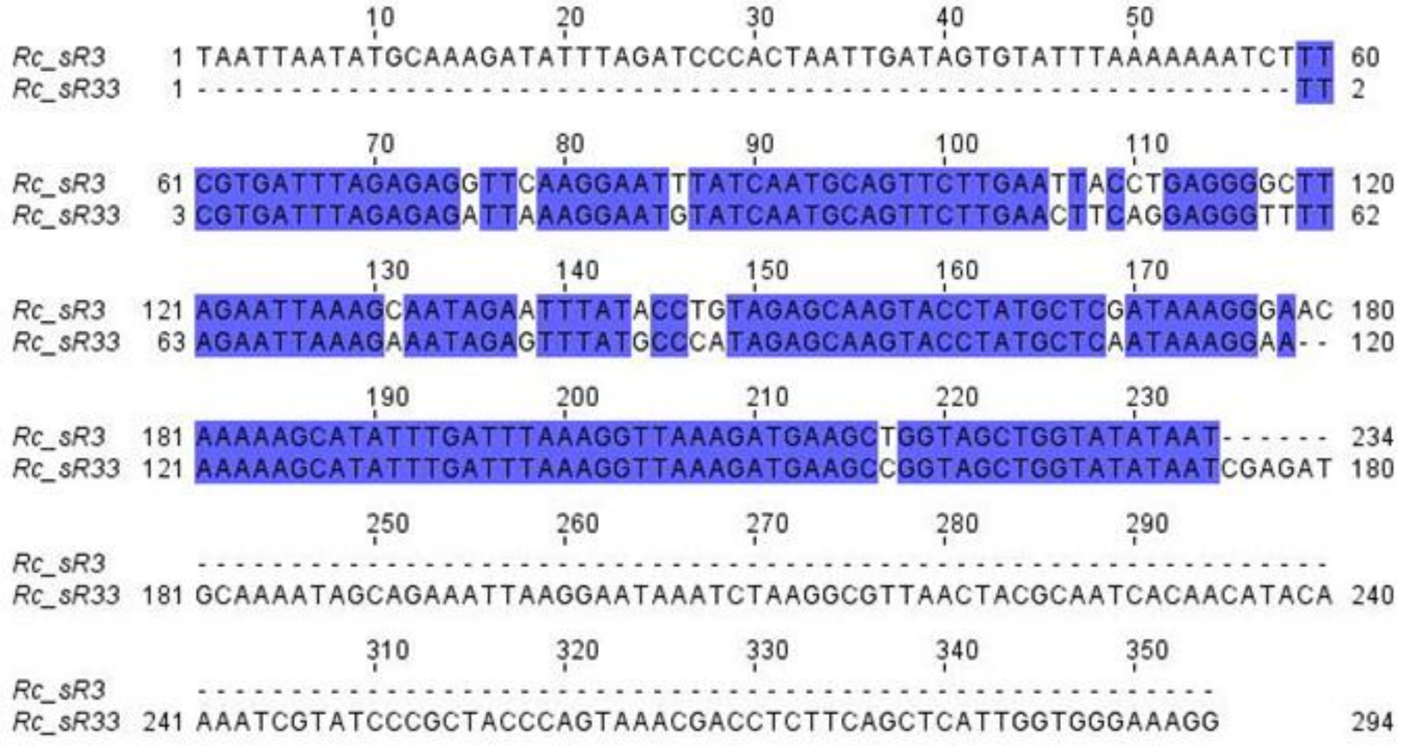

Supplementary Figure S4: Synteny of *RC1218* and its respective adjacent genes in different rickettsial species.

The *R. conorii RC1218* gene (represented by violet arrow), coding for a hypothetical protein, is identified to transcribe *Rc*_sR38 sRNA on the anti-sense strand of the reading frame. The syntenic mapping revealed that despite being conserved in several rickettsial species, including some strains of *R. prowazekii* and *R. typhi*, *RC1218* is independently lost in some of *R. prowazekii* strains. All *R. prowazekii* and *R. typhi* strains were included, but only a representative strains of *R. rickettsii* (Iowa and Sheila Smith), *R. canadensis* (McKiel) and *R. bellii* (OSU 85) were selected for syntenic mapping.

Supplementary Figure S4

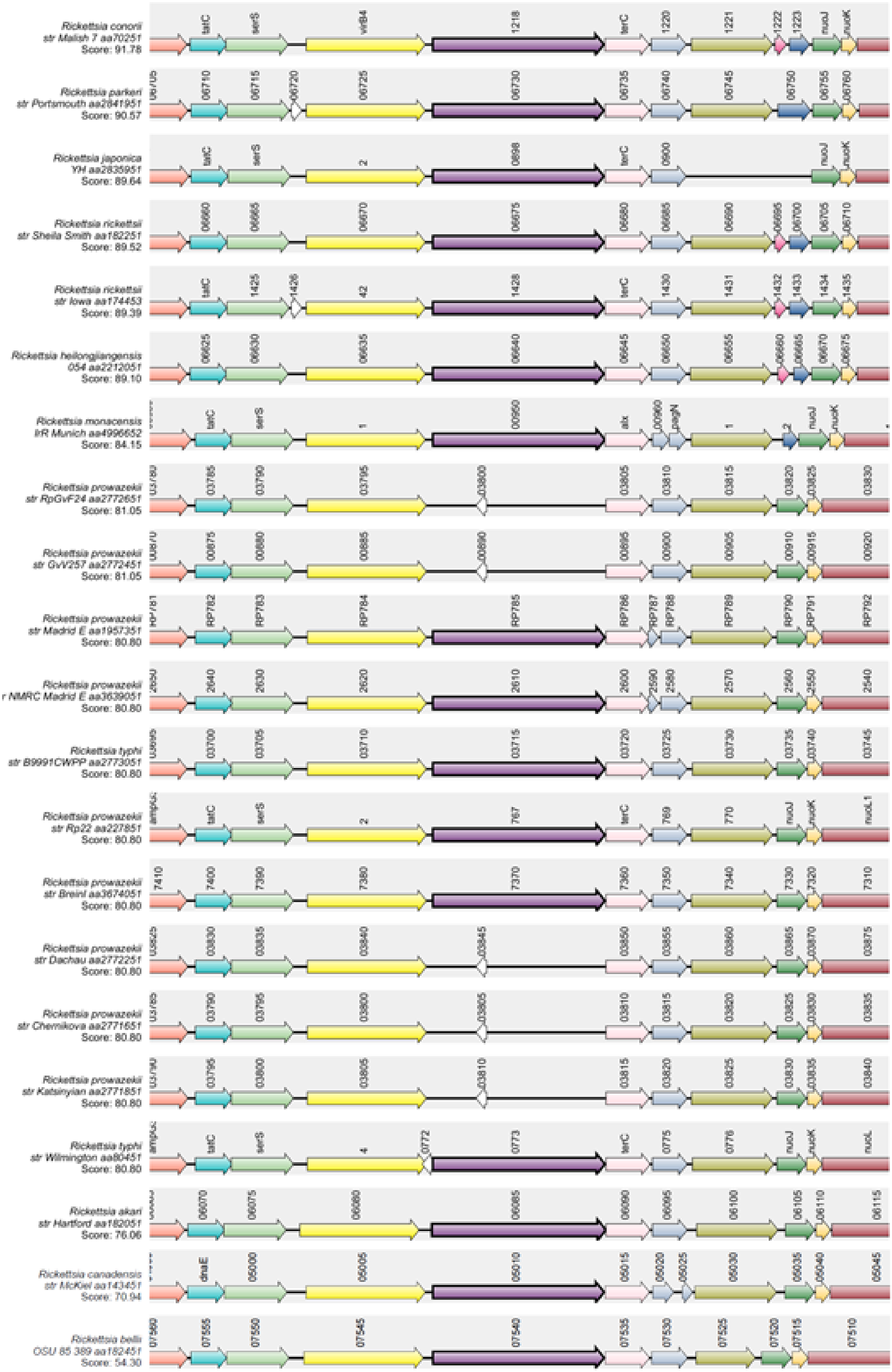

Supplementary Figure S5: **Synteny of *RC1121-RC1122* and their respective adjacent genes in different rickettsial species.**

The *R. conorii RC1121* ORF (pink arrow) and *RC1122* (blue arrow), coding for hypothetical proteins, are identified to transcribe *Rc*_sR34 sRNA on the anti-sense strand of their reading frame and spanning across their intergenic region. The syntenic mapping of *RC1121-1122* revealed that both of these ORFs are unique to *R. conorii* genome (except for *R. slovaca*). Sequence homology of the ORFs to *dnaK* in several other rickettsial genomes was evident and truncated ORFs of *dnaK* were seen in *R. belli* (ancestral group) and *R. akari* (transitional group). Homologous sequences of *RC1121-1122* were absent in all *R. prowazekii* strains but found in *R. typhi* strains. The genomic location of *Rc*_sR34 sRNA on *R. conorii* genome is shown by a red arrow.

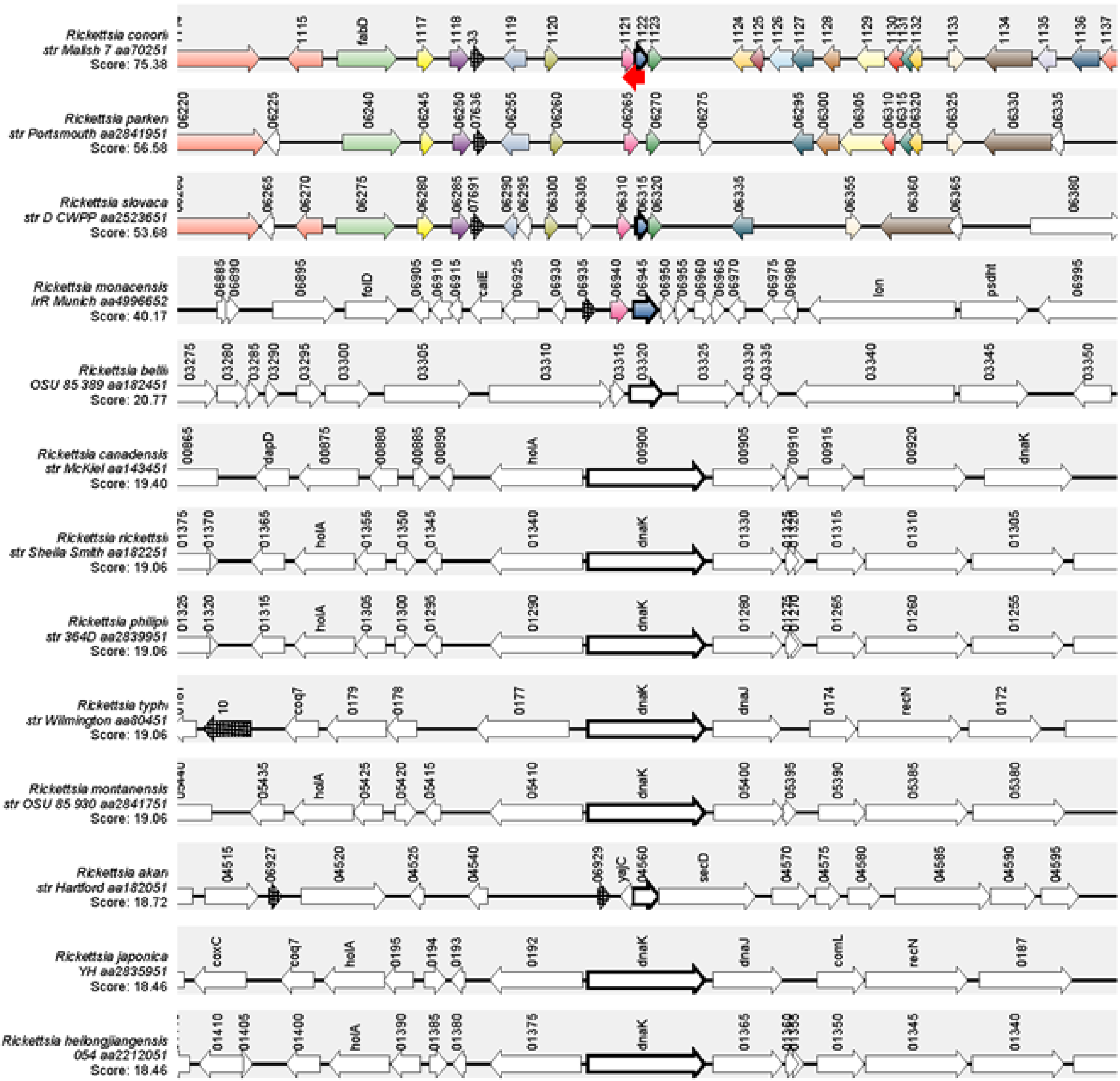

Supplementary Figure S6: **Expression profile of *R. conorii* novel sRNA *Rc*_sR42 and the upstream (*RC1255*) and downstream (*RC1256*) genes adjacent to *Rc*_sR42 during the infection of HMECs *in vitro*.**
The orientation and genomic location of *Rc*_sR42 are shown in the box above and correspond to the PATRIC *R. conorii* genome annotation (PATRIC genome ID: 27944.4). The data from three independent experiments is presented as mean±SEM. Legend: Green: *RC1255*; Red: *Rc*_sR42; Blue: *RC1256*. *= p≤0.05, **=p≤0.01 and ***=p≤0.001.

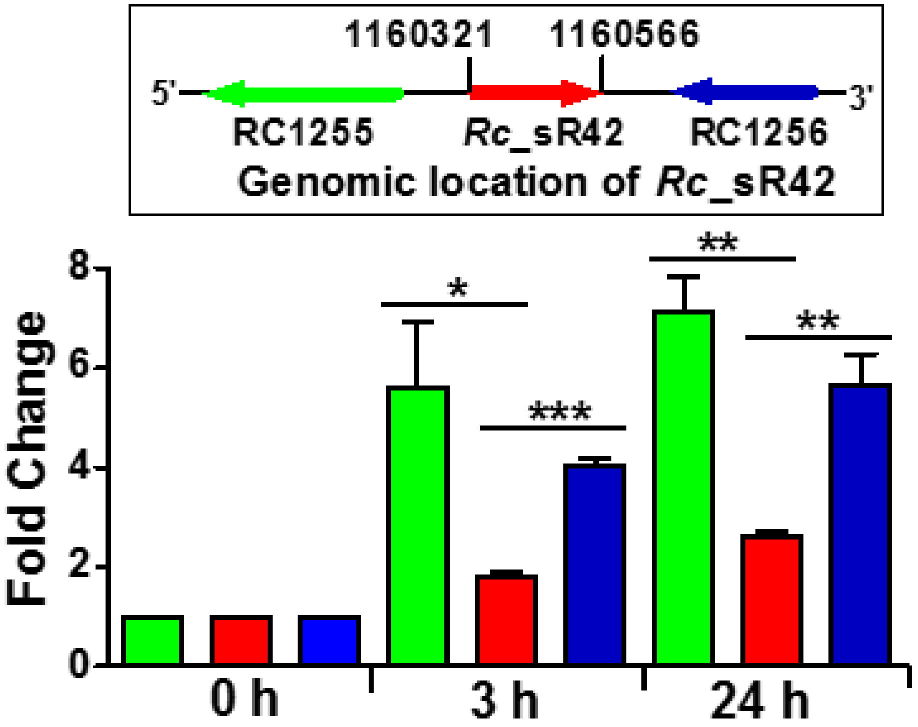

Supplementary Figure S7: **Genomic location of *R. conorii* trans-acting sRNAs *Rc*_sR3 (A) and *Rc*_sR33 (B), and synteny of their respective adjacent genes in different rickettsial species.**

A: Synteny of *Rc*_sR3 and its neighboring genes in different rickettsial species. As shown in the image, the genomic organization both upstream and downstream with respect to *Rc*_sR3 is highly conserved (except for *R. montanensis* and *R. monacensis*) in all rickettsial genomes belonging to spotted fever group. The location and orientation of *Rc*_sR3 sRNA on *R. conorii* genome is shown by a red arrow.

B: Synteny of *Rc*_sR33 and its neighboring genes present in different rickettsial species. While *R. conorii* and *R. parkeri* genomes had conserved synteny, all other rickettsial genomes exhibited diversity in their genome organization both up and downstream of *Rc*_sR33 genomic location. The upstream ORF (*RC1120*), indicated by dark green arrow, was present in only 5 rickettsial genomes (*R. conorii*, *R. parkerii*, *R. slovaca*, *R. montanensis* and *R. monacensis*). The location and orientation of *Rc*_sR33 sRNA on *R. conorii* genome is shown by a red arrow.

Supplementary Figure S7

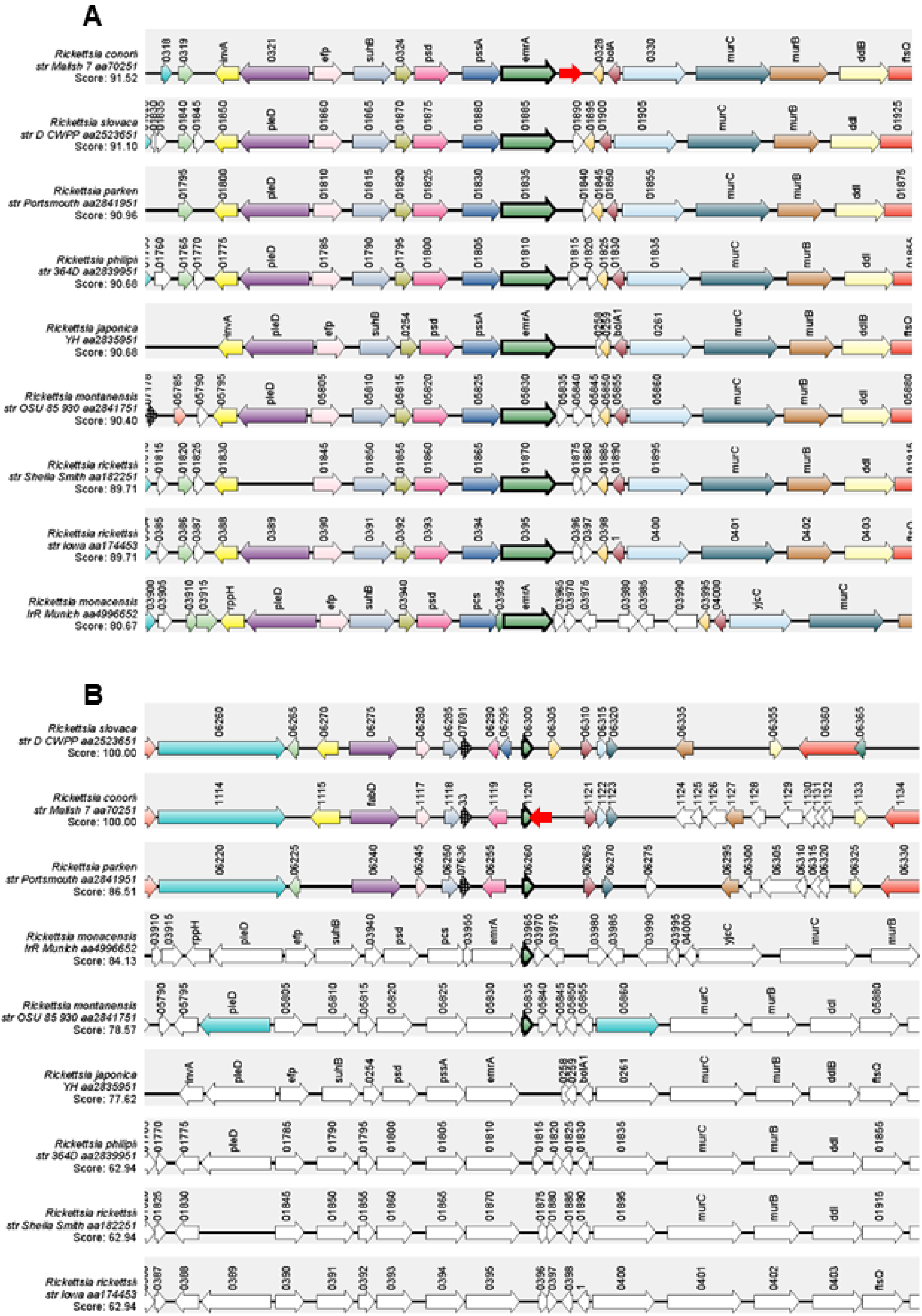

Supplementary Figure S8: **Predicted secondary structure of novel *R. conorii Rc*_sRs.** The minimum free energy secondary structure of *Rc*_sR31, 33, 35, 36 and 42 was determined by RNAfold webserver. The color represents base-pairing probabilities from 0 to 1 (purple to red).

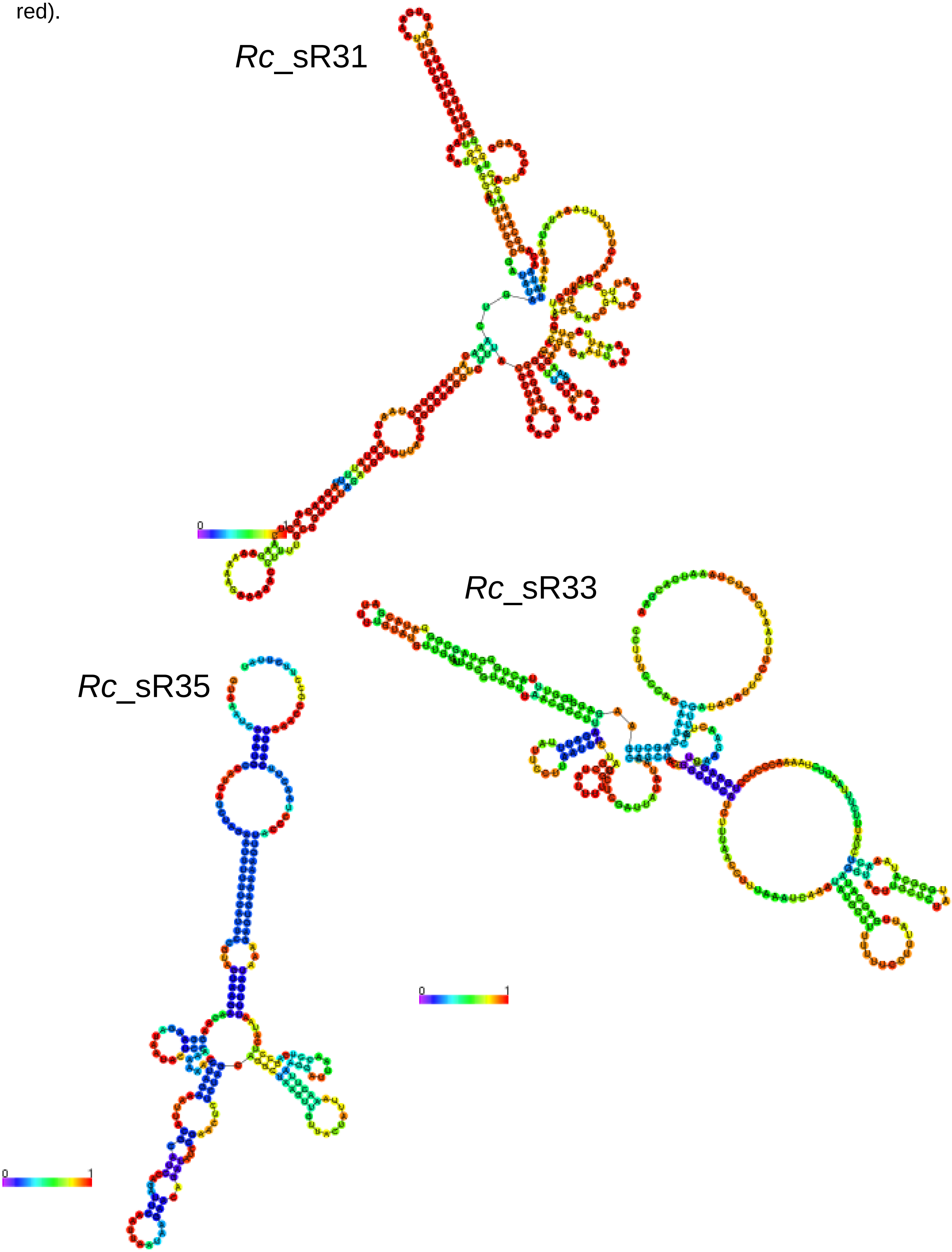

*Rc*_sR36 (6S)

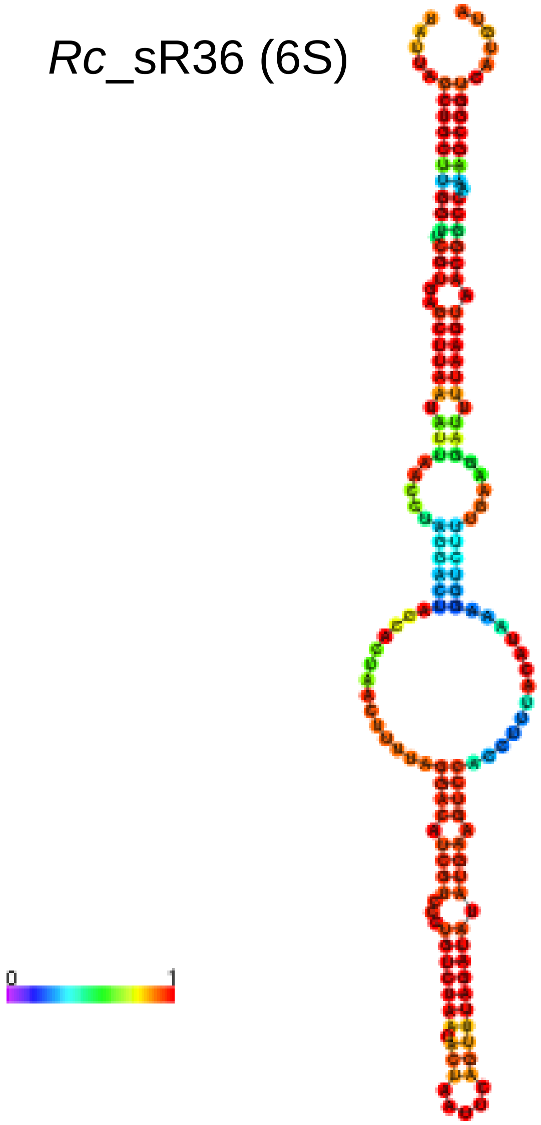

*Rc*_sR42

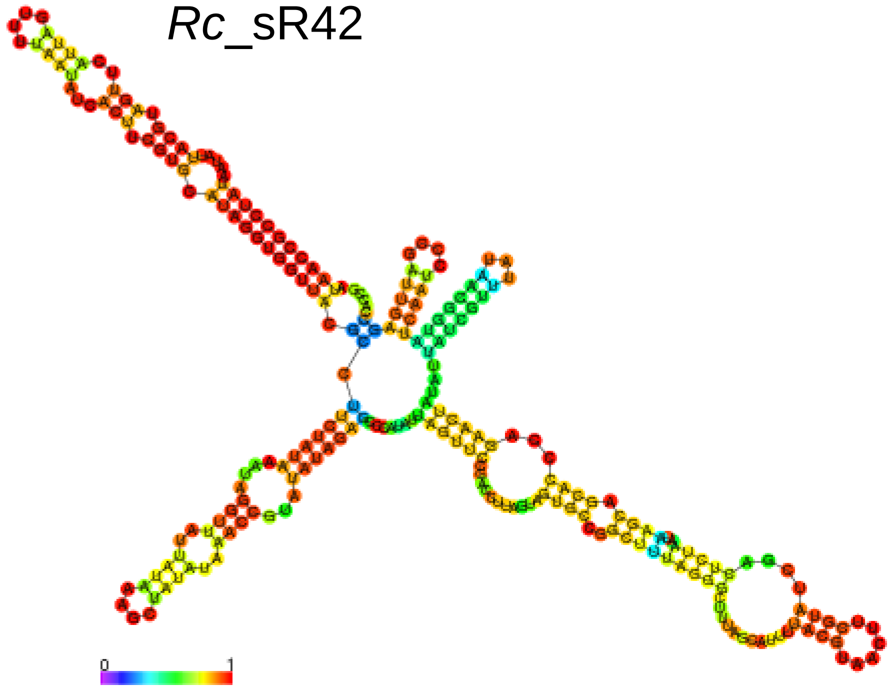

Supplementary Figure S9: Schematic showing the *RC0288* (*cydA*) mRNA fragment used for identification of seed region binding to *Rc*_sR42. (a) Schematic bar diagram showing the *cydA-cydB* genes and their orientation in *R. conorii* genome. The gene co-ordinates shown in brackets correspond to RAST annotation of *R. conorii* genome (PATRIC genome ID: 27944.4). The genomic fragment amplified for EMSA studies is shown as black box. (b) Schematic showing different fragments amplified for the identification of seed region by EMSA studies. The location and orientation of primers on the genomic fragment are shown by black arrows. The 'T7 promoter', if present in the primer is shown as green bar. The IntaRNA predicted seed region is shown by green box. The length of each amplicon is shown on the right hand side. The numbers 1-6 on the left side correspond to the fragment used for EMSA studies presented in Figure 6c. All primer sequences are provided in Supplementary Table S2 online.

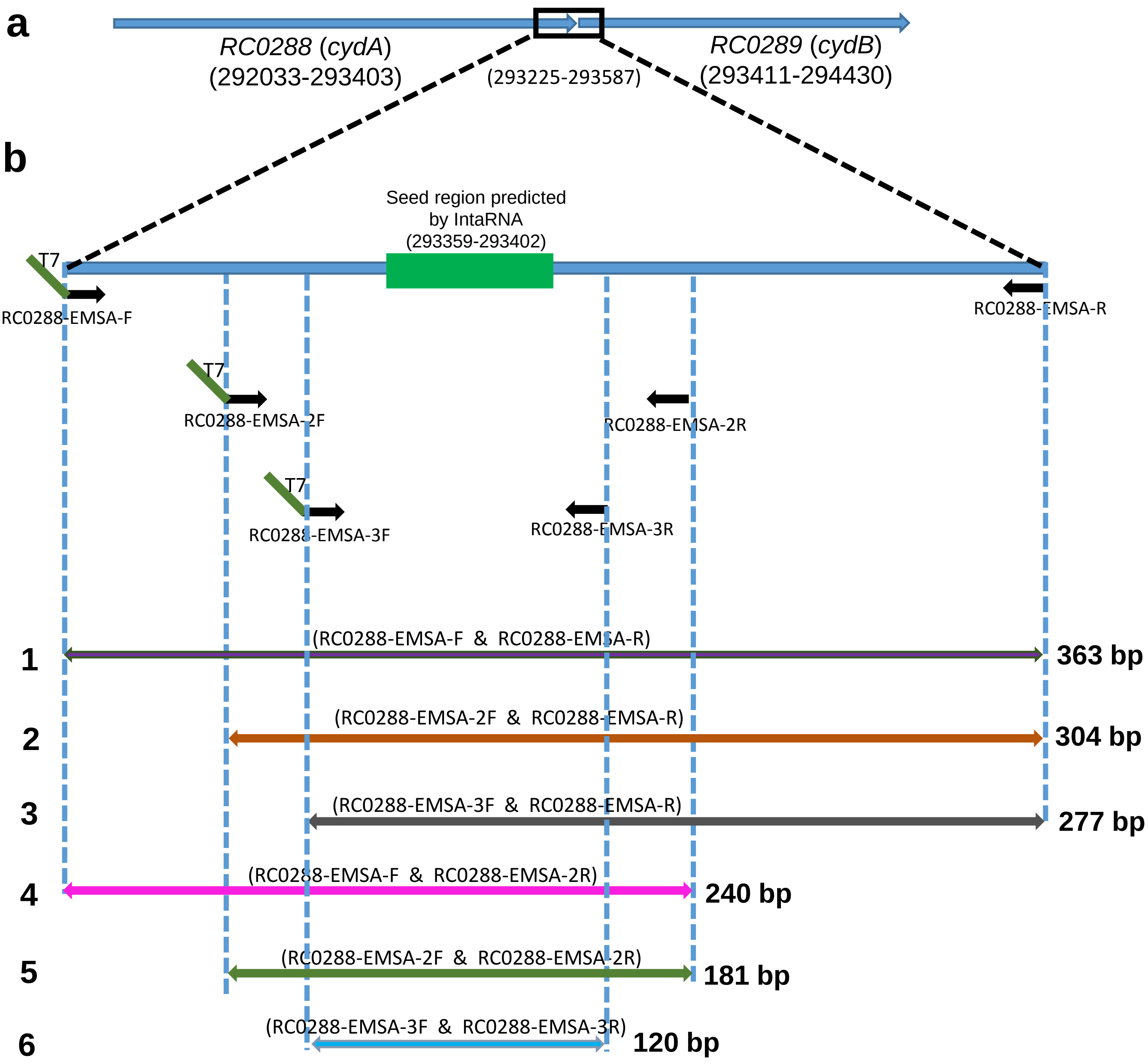

Supplementary Figure S10: **Predicted secondary structure of *R. conorii* riboswitches.** The minimum free energy secondary structure of *Rc*_sR3, 15, 28 and 30 was determined by RNAfold webserver. The color represents base-pairing probabilities from 0 to 1 (purple to red).

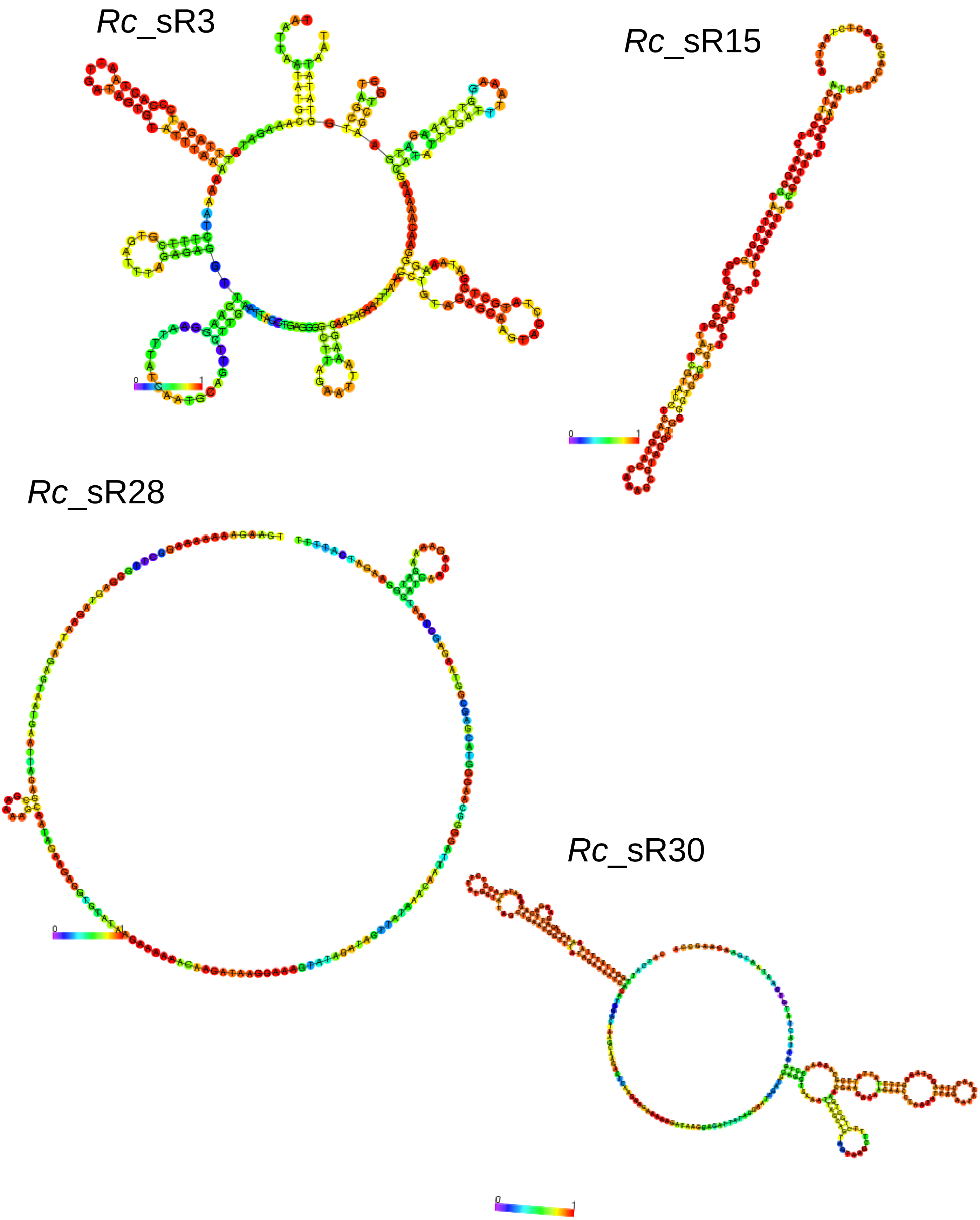

Supplement: Supplementary Information [file srep36728-s1.pdf]
